# Hepatocyte Deletion of IGF2 Prevents DNA Damage and Tumor Formation in Hepatocellular Carcinoma

**DOI:** 10.1002/advs.202105120

**Published:** 2022-05-26

**Authors:** Deepak Kumar, Manasi Das, Alexis Oberg, Debashis Sahoo, Panyisha Wu, Consuelo Sauceda, Lily Jih, Lesley G. Ellies, Magda T. Langiewicz, Supriya Sen, Nicholas J. G. Webster

**Affiliations:** ^1^ Research and Development Service VA San Diego Healthcare System San Diego CA 92161 USA; ^2^ Division of Endocrinology and Metabolism, Department of Medicine University of California San Diego La Jolla CA 92093 USA; ^3^ Division of Genome Information Sciences, Department of Pediatrics University of California San Diego La Jolla CA 92093 USA; ^4^ Division of Cancer Biology Research, Department of Pathology University of California San Diego La Jolla CA 92093 USA; ^5^ Moores Cancer Center University of California San Diego La Jolla CA 92093 USA

**Keywords:** insulin‐like growth factor, liver cancer, prognosis, RNA splicing

## Abstract

Hepatocellular carcinoma (HCC) is the fifth most common cancer worldwide. Serine‐arginine rich splicing factor 3 (SRSF3) plays a critical role in hepatocyte function and its loss in mice promotes chronic liver damage and leads to HCC. Hepatocyte‐specific SRSF3 knockout mice (SKO mice) also overexpress insulin‐like growth factor 2 (IGF2). In the present study, double deletion of Igf2 and Srsf3 (DKO mice) prevents hepatic fibrosis and inflammation, and completely prevents tumor formation, and is associated with decreased proliferation, apoptosis and DNA damage, and restored DNA repair enzyme expression. This is confirmed in vitro, where IGF2 treatment of HepG2 hepatoma cells decreases DNA repair enzyme expression and causes DNA damage. Tumors from the SKO mice also show mutational signatures consistent with homologous recombination and mismatch repair defects. Analysis of frozen human samples shows that SRSF3 protein is decreased sixfold in HCC compared to normal liver tissue but SRSF3 mRNA is increased. Looking at public TCGA data, HCC patients having high SRSF3 mRNA expression show poor survival, as do patients with alterations in known SRSF3‐dependent splicing events. The results indicate that IGF2 overexpression in conjunction with reduced SRSF3 splicing activity could be a major cause of DNA damage and driver of liver cancer.

## Graphical abstract

Deletion of insulin‐like growth factor 2 (IGF2) prevents liver cancer in the serine‐arginine rich splicing factor 3 (SRSF3) knockout mouse model by restoring DNA repair enzymes and preventing DNA damage. SRSF3 protein is reduced in human HCC samples and SRSF3‐dependent splicing predicts survival. IGF2 overexpression and SRSF3 loss could thus be a major cause of DNA damage and liver cancer.

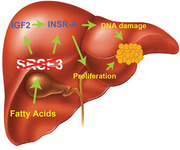

## 1 Introduction

Analysis of the Cancer Genome Atlas has identified alterations in RNA splicing in hepatocellular carcinoma (HCC) and many prognostic splicing signatures have been developed.^[^ ^1^ ^]^ Furthermore, alterations in splicing factor expression have also been observed in HCC and other cancers.^[^ ^1^, ^2^ ^]^ Previously we reported that mice with hepatocyte‐specific deletion of splicing factor SRSF3 (serine‐arginine rich splicing factor 3) (SKO mice) developed spontaneous HCC with aging.^[^ ^3^ ^]^ SRSF3 levels are reduced in early liver disease, including non‐alcoholic fatty liver disease (NAFLD), non‐alcoholic steatohepatitis (NASH), and cirrhosis and in NASH‐associated HCC in humans.^[^ ^4^ ^]^ In the case of hepatitis B virus (HBV)‐associated liver cancer, SRSF3 is sequestered in the cytoplasm, and hence functionally inhibited, by the viral HBx protein.^[^ ^5^ ^]^ SRSF3‐dependent splicing can be also inhibited by alternative mechanisms as the protein phosphatase PPM1G is overexpressed in HCC and dephosphorylates SRSF3 to reduce its ability to regulate alternative splicing.^[^ ^6^ ^]^ The splicing of SRSF3‐target genes is also dysregulated in HCC and, not surprisingly, many of these genes play a role in cell cycle regulation and cancer.^[^ ^5^, ^7^ ^]^

Hepatocyte deletion of SRSF3 in mice resulted in the induction of insulin‐like growth factor 2 (*Igf2)*and *H19* expression.^[^ ^3b^ ^]^ In humans, 20 to 25% of HCCs show elevated *IGF2* mRNA expression that correlates with shorter survival.^[^ ^8^ ^]^ Increased IGF2 protein is seen in HCC tumors and serum IGF2 levels are significantly higher in HCC patients.^[^ ^9^ ^]^ Serum from chronic HBV‐infected patients also contains high levels of IGF2 which can stimulate HCC proliferation.^[^ ^10^ ^]^ Fetal expression of *IGF2* is mediated through proximal promoters P3 and P4 but expression from these is epigenetically silenced in the adult and liver‐specific expression in the adult is driven by a distal promoter P1. The distal P1 promoter is silenced ^[^ ^11^ ^]^ in HCC but the proximal P3 promoter is demethylated and reactivated.^[^ ^12^ ^]^ This promoter switching has also been reported in HBV infection as the HBx protein induces *Igf2* expression from the P3 promoter through demethylation.^[^ ^13^ ^]^ Transcripts derived from P3 and P4 contain a 5' untranslated region (UTR) that is a target for regulation by the IGF2BP/IMP RNA‐binding proteins which increase translation.^[^ ^14^ ^]^ These IGF2BPs are also overexpressed in HCC,^[^ ^15^ ^]^ so these transcripts may lead to higher IGF2 protein levels. This promoter switching may be important in HCC as altering the methylation status of P4 to reduce *IGF2* expression enhances survival in a Hep3B liver cancer model ^[^ ^16^ ^]^ and reducing *IGF2* levels by siRNA reduces c‐Myc and N‐Ras signaling in HCC.^[^ ^17^ ^]^

Normally the liver does not respond to IGF2 as IGF1R expression is very low and the insulin receptor (INSR) only expresses the B isoform (INSR‐B exon 11 included), which does not bind IGF2.^[^ ^18^ ^]^ Loss of SRSF3, however, causes expression of the INSR‐A (exon 11 skipped) isoform that binds IGF2 with high affinity.^[^ ^3b^ ^]^ So, in this study, we investigated whether the elevated *Igf2* expression seen when hepatocyte SRSF3 levels are reduced contributes to the carcinogenic phenotype through activation of INSR‐A. We found that IGF2 expression is essential for liver fibrosis, inflammation, and tumor formation by triggering DNA damage, apoptosis, and proliferation. Through analyzing the TCGA data, we also found that patients with high expression of *IGF2* have worse overall survival, and that the switch to using the proximal promoter P3 is associated with higher *IGF2* expression. Furthermore, we found that patients with alterations in SRSF3‐target gene splicing also have worse survival, supporting the importance of the SRSF3/IGF2/INSR‐A axis in HCC.

## 2 Results

### 2.1 Hepatocyte Specific Deletion of IGF2 Prevented HCC Development in a Mouse Model of HCC

**Figure 1:**
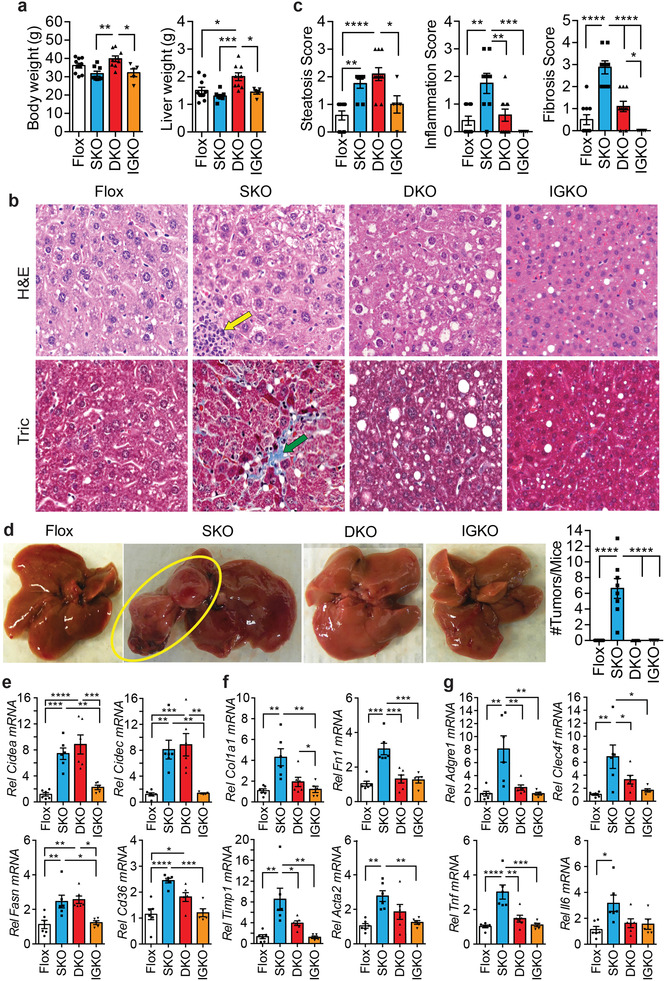
Genetic loss of *Igf2* prevents HCC in hepatocyte‐specific *Srsf3* knockout mice. a) Body weight and liver weight in *Srsf3*‐HKO (SKO, blue), *Srsf3‐Igf2*‐HKO (DKO, red), *Igf2*‐HKO (IGKO, orange), and control (Flox, white) mice. Individual animals are shown. Bars show mean ± SEM. b) Fixed liver sections stained with hematoxylin and eosin (H&E) or Masson's Trichrome (Tric). Scale bar indicates 50 µm. Yellow arrow indicates an inflammatory infiltrate. Green arrow indicates a region of fibrosis. c) Steatosis score, inflammation score, and fibrosis score for the four genotypes. d) Gross morphology of the livers from the four genotypes. Yellow circle highlights two tumors. Number of tumors per mouse is quantified in the bar graph to right. e) Expression of lipid storage and synthesis genes, cell death‐inducing DFFA‐like effector A (*Cidea)*, cell death‐inducing DFFA‐like effector C/FSP27 (*Cidec)*, fatty‐acid synthase (*Fasn)*, and fatty‐acid translocase (*Cd36)*, in livers from Flox (white), SKO (blue), DKO (red), and IGKO (orange) mice. f) Expression of fibrosis related genes, collagen 1A1 (*Col1a1)*, fibronectin (*Fn1)*, tissue inhibitor of metalloproteinases 1 (*Timp1)*, and smooth muscle actin (*Acta2)*, in livers from Flox (white), SKO (blue), DKO (red), and IGKO (orange) mice. g) Expression of inflammation‐associated genes, adhesion G protein‐coupled receptor E1/EMR1 (*Adgre1*), C‐type lectin domain family 4f *(Clec4f)*, tumor necrosis factor (*Tnf)*, and interleukin‐6 (*Il6)*, in livers from Flox (white), SKO (blue), DKO (red), and IGKO (orange) mice. For all graphs, bars show mean ± SEM and asterisks show statistical significance by ANOVA; *p < 0.05, **p < 0.01, ***p < 0.001, and ****p < 0.0001 for the indicated comparison.

As SRSF3‐HKO (SKO) mice show liver damage and HCC, we are interested in whether hepatocyte deletion of IGF2 would rescue the age‐associated carcinogenic phenotype. We generated a hepatocyte‐specific double deletion of SRSF3 and IGF2 (DKO), or single IGF2 deletion (IGKO) mice. This deletion was specific for *Igf2* and did not affect expression of the *H19* gene (Figure S1, Supporting Information). The body weight and liver weight of 12‐month‐old DKO mice were greater than either single deletion SKO and IGKO mice (**Figure** **1**a). There was no difference in percentage liver to body weight, or lung weight, (Figure S2a,b, Supporting Information), however, the weights of visceral fat and pancreas tissue in DKO mice were higher than SKO (Figure S2c,d, Supporting Information), and the spleen was larger in both the SKO and DKO mice (Figure S2e, Supporting Information). Liver sections from SKO and DKO mice showed marked steatosis, whereas the livers from IGKO mice were comparable to the control Flox mice (Figure 1b,c). SKO mice showed infiltration of inflammatory cells and extensive intralobular fibrosis by Masson's trichrome that was absent from DKO and IGKO mice. Most importantly, the livers of all the SKO mice showed multiple spontaneous HCC tumors while there was no evidence for liver tumors in any of DKO and IGKO mice (Figure 1d). The carcinogenic phenotype was specific for SRSF3 as deletion of the related splicing factor SRSF1 did not cause HCC despite elevated *Igf2* expression (Figure S3a–c, Supporting Information). Interestingly, the SRSF1‐HKO livers did not show alteration of *Insr* splicing and the increased expression of INSR‐A that is seen in the SKO mice (Figure S3d, Supporting Information).

The changes in steatosis, inflammation, and fibrosis were confirmed by QPCR. Livers from the SKO and DKO mice showed high expression of the lipid metabolism genes *Cidea*, *Cidec*, *Fasn*, and *Cd36* (Figure 1e). The SKO mice but not the DKO and IGKO mice showed elevation of the fibrogenic genes *Col1a1*, *Fn1, Timp1*, and *Acta2* (Figure 1f), the macrophage and Kupffer cell markers *Emr1* and *Clec4f*, and the inflammatory cytokines tumor‐necrosis factor alpha (*Tnfa)* and interleukin 6 *(Il6)* (Figure 1g).

Deletion of IGF2 also had mild effects on whole‐body glucose metabolism. Fasting glucose levels were decreased in the SKO mice but they were restored in the DKO mice (Figure S4a, Supporting Information). The SKO mice had lower blood glucose at 0 and 15 min during a glucose tolerance test (Figure S4b, Supporting Information). The DKO mice showed normal insulin tolerance, however, and

quickly restored glucose levels after insulin injection (Figure S4c, Supporting Information). Fasting IGF2 levels were lower in the DKO mice, IGF1 levels were lower in the SKO mice, insulin levels were higher in the DKO mice, but growth hormone levels were unchanged (Figure S4d, Supporting Information). Plasma total cholesterol was unchanged, but HDL cholesterol was lower in the SKO mice, and triglycerides were higher in the DKO than SKO (Figure S4e, Supporting Information).

### 2.2 IGF2 Deletion Attenuated Proliferation, Apoptosis, and DNA Damage in SRSF3‐HKO Mice

**Figure 2:**
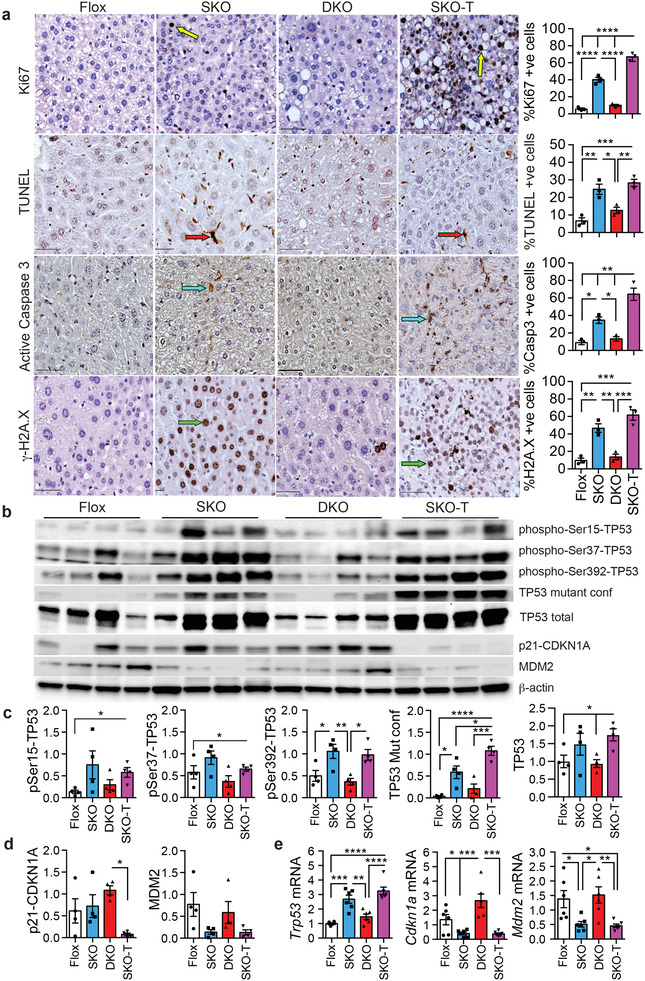
Genetic loss of *Igf2* inhibits hepatocyte proliferation, apoptosis, and DNA damage. a) Staining for proliferation by Ki67, apoptosis by TUNEL and cleaved (active) caspase 3, or double‐stranded DNA breaks by *γ*‐H2A.X in liver sections from Flox, SKO, and DKO mice, and from tumor sections from SKO mice (SKO‐T). Positive nuclei or cells are labeled brown. Typical Ki67 positive cells are indicated by yellow arrows, TUNEL positive cells by red arrows, active caspase 3 positive cells by blue arrows, and *γ*‐H2A.X positive cells by green arrows. Graphs on right show quantification from three sections per mouse for three mice per group: Flox (white), SKO (blue), DKO (red), and SKO‐T (magenta). b) Immunoblots of liver extracts from Flox, SKO, and DKO livers and SKO tumors (SKO‐T). Extracts were blotted for tumor suppressor p53 (TP53) phosphorylated on Ser15, Ser 37, or Ser392, mutant conformation TP53, total TP53, cyclin‐dependent kinase inhibitor 1A (p21/CDKN1A), mouse double minute 2 (MDM2), and *β*‐actin as a loading control. c) Quantification of TP53 immunoblotting from panel E. d) Quantification of CDKN1a and MDM2 immunoblotting from panel E. e) Trp53, Cdkn1a, and Mdm2 mRNA expression by qPCR. For all graphs, bars show mean ± SEM and asterisks show statistical significance by ANOVA; *p < 0.05, **p < 0.01, ***p < 0.001, and ****p < 0.0001 for the indicated comparison.

The previous results suggested that IGF2 was promoting tumorigenesis in the SKO mice as the DKO did not form any tumors. Sections from the SKO liver and tumors (SKO‐T) showed elevated Ki67 staining, elevated TUNEL positive cells, and increased cleaved Caspase 3 staining (**Figure** **2**a). The sections from DKO livers did not show the increased staining for these markers indicating that loss of IGF2 prevented proliferation and apoptosis of hepatocytes. SKO livers and tumors also showed evidence of DNA damage by *γ*‐H2A.X staining, a marker of double‐stranded DNA breaks (DSB),^[^ ^19^ ^]^ which was absent in the DKO livers (Figure 2a). Consequently, we assessed p53 phosphorylation on both Ser15 and Ser37, which are DNA damage‐induced sites,^[^ ^20^ ^]^ and both were elevated in the SKO and SKO‐T extracts, but not the extracts from DKO mice (Figure 2b,c). Phosphorylation of p53 on Ser392, which is important for mitochondrial translocation and induction of apoptosis,^[^ ^21^ ^]^ was also elevated in SKO livers and tumors, but not the DKO livers. The tissues also showed increased total p53 protein and mutant conformation p53 in the SKO liver and SKO‐T extracts (Figure 2b,c) but not DKO liver extracts. As an assessment of p53 function, p21‐CDKN1A and MDM2 expression was reduced in SKO tumors, and MDM2 expression was reduced in SKO livers (Figure 2b,d). At the RNA level, livers and tumors from SKO also showed increased expression of *Trp53* (p53) and reduced expression of *Cdkn1a* (p21) and *Mdm2* (Figure 2e).

### 2.3 Deletion of SRSF3 Caused DNA Damage and Prevented Its Repair

**Figure 3:**
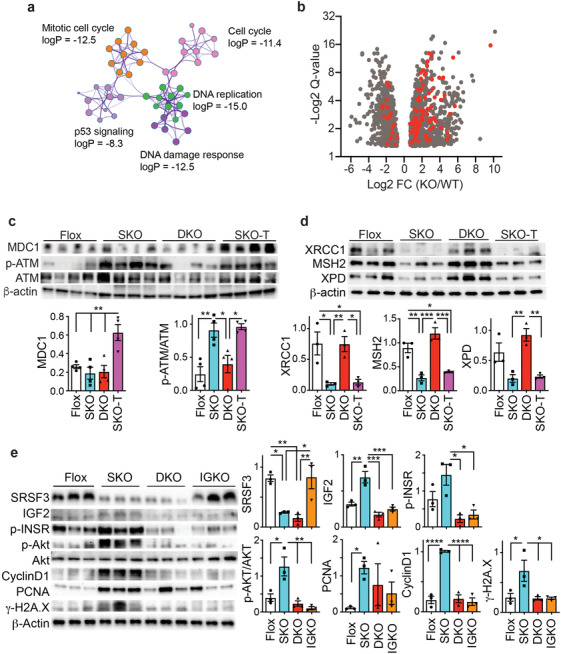
Genetic loss of *Igf2* prevents induction of the DSB DNA‐damage response. a) *Srsf3*‐dependent genes are enriched for a large cluster of gene ontology terms including sub‐clusters involved in mitotic cell cycle (orange), cell cycle (pink), DNA replication (green), DNA damage (dark purple), and p53 signaling (light purple). The log_10_ *p*‐value for enrichment is indicated for each sub‐cluster. b) Volcano plot of SRSF3‐dependent genes (gray dots) showing Log_2_ fold change (KO/WT) versus ‐Log_2_ FDR q‐value. Genes in the cluster in panel A are highlighted in red. c) Immunoblots of liver extracts from Flox, SKO, and DKO livers and SKO tumors (SKO‐T). Extracts were blotted for mediator of DNA damage checkpoint protein 1 (MDC1), ataxia telangiectasia mutated kinase (ATM) phosphorylated on Ser1981, total ATM, and *β*‐actin as a loading control. Graphs show quantification of ATM phosphorylation and MDC1: Flox (white), SKO (blue), DKO (red), and SKO‐T (magenta). d) Immunoblots of liver extracts from Flox, SKO, and DKO livers and SKO tumors (SKO‐T). Extracts were blotted for the DNA repair proteins XRCC1, MSH2, XPD, and *β*‐actin as a loading control. Graphs show quantification: Flox (white), SKO (blue), DKO (red), and SKO‐T (magenta). e) Immunoblots of liver extracts from Flox, SKO, DKO, and IGKO livers. Extracts were immunoblotted for SRSF3, IGF2, phospho‐INSR(Tyr1158,62,63), phospho‐AKT(Ser473), total AKT1, cyclin D1, PCNA, *γ*‐H2A.X, and *β*‐actin as a loading control. Graphs show quantification: Flox (white), SKO (blue), DKO (red), and IGKO (orange). For all graphs, bars show mean ± SEM and asterisks show statistical significance by ANOVA; *p < 0.05, **p < 0.01, ***p < 0.001, and ****p < 0.0001 for the indicated comparison.

Loss of SRSF3 led to increased DNA damage and carcinogenesis in hepatocytes, so we analyzed a previous hepatocyte RNAseq dataset for genes associated with DNA damage and the cell cycle. This dataset was derived from *Srsf3*‐floxed hepatocytes that had been acutely infected with an adenovirus expressing Cre recombinase or green fluorescent protein as a control.^[^ ^4a^ ^]^ Genes whose expression was altered by acute loss of SRSF3 were mapped to gene ontologies and pathways using Metascape.^[^ ^22^ ^]^ Three large clusters of enriched terms were observed; the first contained genes involved in DNA damage, p53 signaling, and cell cycle regulation (**Figure** **3**a), a second contained genes involved in metabolic processes (Figure S5a, Supporting Information), and a third contained genes involved in the hemostatic response of the liver (Figure S5b, Supporting Information). The genes in the first cluster are highlighted in red on the volcano plot (Figure 3b and Table S1, Supporting Information) and the majority show increased expression consistent with increased DNA damage. To test whether loss of SRSF3 could directly cause DNA damage, we knocked down SRSF3 expression in human HepG2 hepatoma cells in vitro and observed increased expression of *γ*‐H2A.X (Figure S5c, Supporting Information). Both livers and tumors from SKO mice showed elevated phosphorylation of ATM on Ser1981 (Figure 3c), a site phosphorylated in response to DSBs.^[^ ^23^ ^]^ The DNA‐damage scaffold protein MDC1, which interacts with ATM,^[^ ^24^ ^]^ was also expressed at a high level in tumors from SKO mice. The extract from livers and tumors from SKO mice showed reduced expression of other DNA damage pathways including XRCC1 (X‐ray repair cross‐complementing protein 1), MSH2 (MutS homolog 2), and XPD (Xeroderma pigmentosum group D) (Figure 3d) indicating that loss of SRSF3 may impair other DNA damage responses at the same time as increasing DSBs.

### 2.4 IGF2 Induces DNA Damage In Vitro

The previous results indicated that loss of IGF2 in the SKO mice prevented DNA damage and activation of the ATM pathway. It also prevented the loss of other DNA damage response proteins. So, we investigated whether IGF2 could affect these DNA damage response pathways in hepatocytes in vitro. We initially examined whether hepatocyte deletion of IGF2 could inhibit INSR/IGF1R signaling, proliferation, and DNA damage in primary hepatocyte cultures from 1‐month old Flox, SKO, DKO, and IGKO mice. Liver *Igf1r* expression is very low level compared to the *Insr* in both mouse and human (Figure S5d, Supporting Information). Expression of SRSF3 and IGF2 was reduced as expected in primary hepatocytes from the respective knockouts (Figure 3e). The SKO mice showed elevated expression of IGF2 and activation of the INSR (phospho‐Tyr1158/62/63) and downstream AKT signaling (phospho‐Ser473) by immunoblotting.^[^ ^25^ ^]^ SKO hepatocytes also showed elevated expression of cyclinD1, PCNA, and *γ*‐H2A.X that was not seen in DKO and IGKO hepatocytes (Figure 3e).

**Figure 4:**
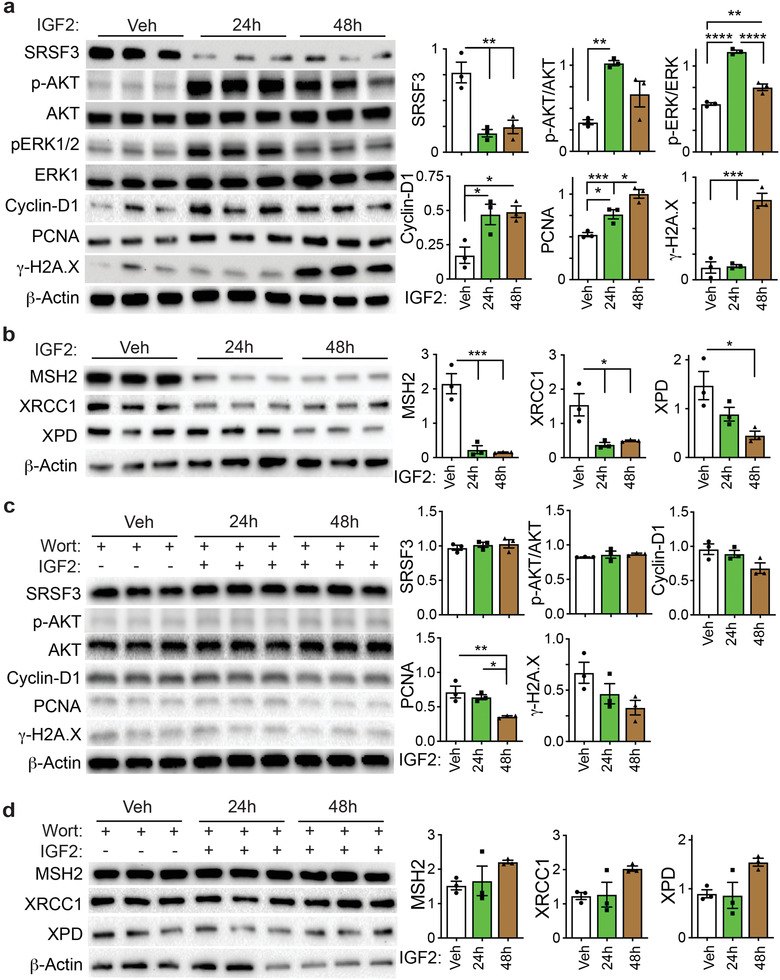
IGF2 activates AKT and ERK signaling, represses SRSF3 expression, and causes DNA damage in HepG2 cells. HepG2 cells were serum starved then stimulated with 100 ng mL^−1^ IGF2 for 24 and 48 h in the a,b) absence or c,d) presence of 50 nm wortmannin. a,b) Cell extracts were immunoblotted for SRSF3, phospho‐AKT(Ser473), total AKT1, phospho‐ERK(Thr202/Tyr204), total ERK1, cyclin D1, PCNA, *γ*‐H2A.X, and *β*‐actin as a loading control. Graphs show quantification: vehicle (white), 24 h (green), and 48 h (brown). c,d) Cell extracts were immunoblotted for MSH2, XRCC1, XPD, and *β*‐actin as a loading control. Graphs show quantification: vehicle (white), 24 h (green), and 48 h (brown). For all graphs, bars show mean ± SEM and asterisks show statistical significance by ANOVA; *p < 0.05, **p < 0.01, ***p < 0.001, and ****p < 0.0001 for the indicated comparison.

To test whether IGF2 could directly affect cell cycle proteins, DNA damage, and DNA damage response proteins, we stimulated human HepG2 hepatoma cells with 100 ng mL^−1^ IGF2 for 24 and 48 h. HepG2 cells express both the INSR‐A and INSR‐B isoforms and the IGF1R. Initially, we investigated the effect of IGF2 on insulin signaling pathways and SRSF3 levels. IGF2 activated AKT (pSer473) and ERK1/2 (pThr202/Tyr204) phosphorylation at 24 and 48 h by immunoblotting (**Figure** **4**a). Unexpectedly, IGF2 stimulation also caused time‐dependent loss of SRSF3. The IGF2 effect was specific for SRSF3 as other SR proteins did not change in expression (Figure S6a, Supporting Information). The reduction in SRSF3 did not seem to be mediated through proteosomal degradation as it still occurred in the presence of MG132 (Figure S6b, Supporting Information). mRNA expression for both the full‐length and truncated form of *SRSF3* increased at 48 h consistent with the known autoregulation (Figure S6c, Supporting Information) indicating that the decrease was not mediated at the transcriptional level. We then measured the expression of cell cycle proteins and *γ*‐H2A.X for DNA damage. IGF2 stimulation increased cyclin‐D1 and PCNA expression at 24 and 48 h and increased *γ*‐H2A.X levels at 48 h (Figure 4a). As loss of SRSF3 caused loss of DNA‐damage response protein expression in liver extracts (Figure 3d), we tested whether IGF2 stimulation would also cause loss of DNA repair enzymes. IGF2 stimulation of HepG2 cells reduced expression of XRCC1, MSH2, and XPD (Figure 4b). Pretreatment of cells with 50 nm wortmannin for 30 min blocked all IGF2 effects suggesting a role for PI‐3Kinase signaling (Figure 4c,d).

### 2.5 Deletion of IGF2 Restores Binuclear Tetraploid Liver Cells

**Figure 5:**
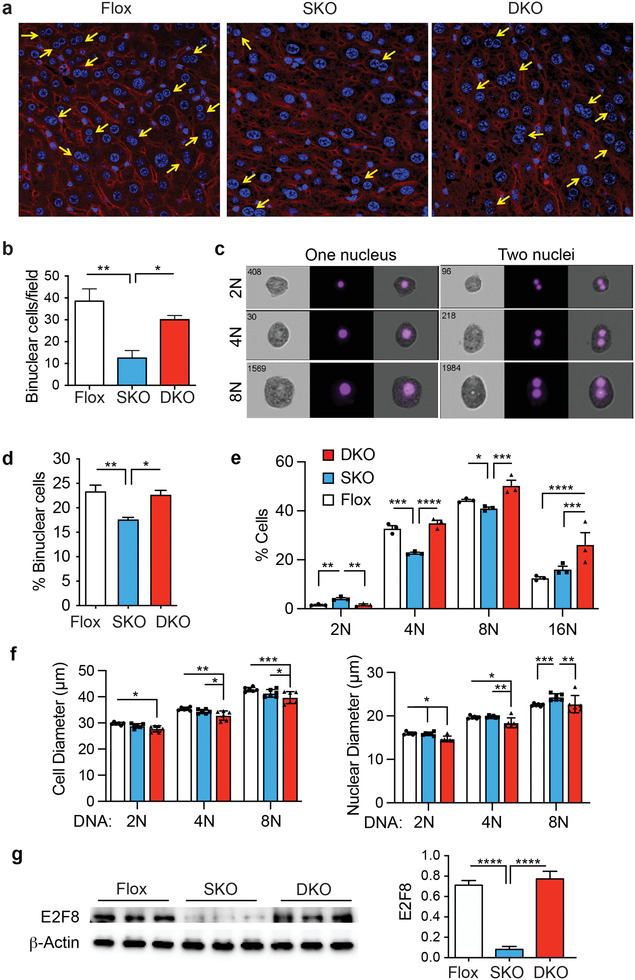
Genetic loss of Igf2 restores hepatocyte polyploidy and binuclearity. a) Liver sections from Flox, SKO, and DKO livers were stained with DAPI (blue) to identify nuclei and for *β*‐catenin (red) to identify the cell plasma membrane. Yellow arrows indicate binuclear cells. b) Graph shows quantification of binuclear cells from the fluorescence images: Flox (white), SKO (blue), and DKO (red). c) Representative images of diploid (2N), tetraploid (4N), and octaploid (8) nuclei in mononuclear and binuclear cells from imaging flow cytometry. Left panel shows bright‐field image, central panel shows DAPI fluorescence, right panel shows superposition. d) Quantification of binuclear cells from imaging flow cytometry: Flox (white), SKO (blue), and DKO (red). e) Graph showing percentage of cells with diploid (2N), tetraploid (4N), octaploid (8N), and hexadecaploid (16N) DNA content. f) Cell diameter and nuclear diameter of cells with diploid (2N), tetraploid (4N), and octaploid (8N) DNA content. g) Immunoblotting of liver extracts from Flox, SKO, and DKO mice for endoreplication factor E2F8 and for *β*‐actin as loading control. Graph shows quantification. For all graphs, bars show mean ± SEM and asterisks show statistical significance by ANOVA; *p < 0.05, **p < 0.01, ***p < 0.001, and ****p < 0.0001 for the indicated comparison.

The liver plays a crucial role in detoxifying environmental contaminants and metabolites. Hepatocyte polyploidy helps minimize potentially deleterious effects of these metabolites to cause DNA damage.^[^ ^26^ ^]^ Polyploidy is an important feature of mammalian hepatocyte maturation, and insulin signaling increases tetraploidy and binuclearity by AKT‐mediated inhibition of cytokinesis.^[^ ^27^ ^]^ We previously reported that SKO livers show decreased polyploid hepatocytes and reduced binuclearity.^[^ ^3a^ ^]^ Therefore, nuclearity was assessed in the DKO livers by DAPI staining and immuno‐staining for *β*‐catenin to highlight the cell plasma membrane. As expected, sections from SKO mice had fewer binuclear cells compared to flox controls but sections from the DKO livers had normal numbers of binuclear cells (**Figure** **5**a,b). Further to confirm our staining results, we used imaging flow cytometry to analyze primary hepatocytes from SKO, DKO, and flox mice (Figure 5c). Flow cytometry confirmed the reduced number of binuclear cells in SKO livers (Figure 5d). The flow cytometry also showed that SKO livers had increased diploid cells and decreased tetraploid and octoploid cells as compared to DKO and flox livers (Figure 5e). As expected, the cell and nuclear diameters increased with increasing DNA content, but the DKO cells and nuclei were significantly smaller than the other groups (Figure 5f).

E2F8 is a transcriptional repressor of the E2F1 family that regulates cell cycle progression by repressing E2F driven transcription during S‐phase through a negative feedback loop.^[^ ^28^ ^]^ Recent studies have suggested that E2F8 is important for terminal endoreplication and polyploidy in mammalian cells and its loss reduces binuclearity and the formation of polyploid nuclei.^[^ ^29^ ^]^ E2F8 has also been shown to function as both a tumor suppressor during early liver development and an oncogene in HCC.^[^ ^30^ ^]^ So, we compared the expression of E2F8 in liver extracts from the three mice strains and found that E2F8 levels are reduced in the SKO liver but restored in the DKO liver consistent with the restored polyploidy (Figure 5g).

### 2.6 Tumors from SRSF3‐HKO Mice Show Mutational Signatures Consistent with Defects in DNA Repair

**Figure 6:**
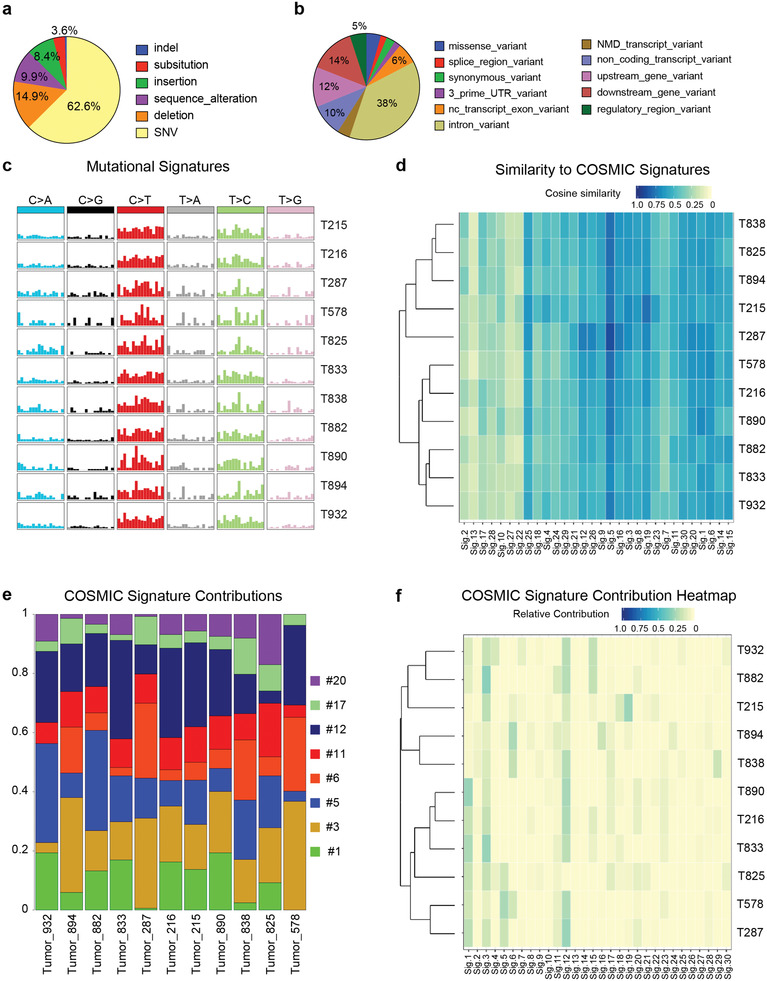
Tumors in SKO mice show mutational signatures consistent with defects in homologous recombination and DNA damage repair. a) Pie chart showing proportion of variant classes found in exome sequencing of 11 SKO tumors (T215‐T932) compared to matched normal tissue. b) Pie chart showing predicted consequence of exome variants. c) Mutational profiles found in 11 SKO tumors. d) Similarity of tumor signatures to known human signatures taken from the COSMIC database. Comparison based on cosine similarity. Color indicates degree of similarity (1 = identical, 0 = no similarity). e) Deconvolution of mouse mutational signatures into human COSMIC signatures. Only signatures contributing >3% are shown. f) Heatmap showing contribution of all 30 COSMIC signatures to the individual tumor profiles. Color indicates relative contribution.

To assess whether the DNA damage observed in the SKO mice leads to tumorigenesis, we performed exome sequencing on 11 tumors and assessed sequence variation and mutational profile (Table S2, Supporting Information). Tumors had varying numbers of variants, from 7398 to 12 157, and ≈72% of these were novel. The major class of variant was single nucleotide variants (SNVs) with smaller numbers of indels, substitutions, deletions, insertions, or other sequence alterations (**Figure** **6**a). As expected, the variants mapped mainly to intronic regions (38%) followed by upstream and downstream gene variants (12 and 14%) and variants in non‐coding RNAs (ncRNAs) and in exons of ncRNAs (10 and 6%) (Figure 6b). The tumor mutational profiles (Figure 8C) clustered into groups of 4 and 7 tumors (Figure S7a, Supporting Information). We analyzed the variants for mutational signatures (Figure S7b, Supporting Information). Two signatures explained >90% of the variance (Figure S7c, Supporting Information), and 6 signatures explained >98% (Figure S7d, Supporting Information). To compare these to known mutational profiles, we analyzed the data for signatures from the COSMIC database. The individual tumor profiles were compared to COSMIC signatures using cosine similarity. All the tumors showed greatest similarity to Signature 5 (Figure 6d), which is a clock‐like aging signature, then to Signatures 25, 12, 6, and 19. We analyzed the relative contributions of mutational signatures that account for greater than 3% of the profile (Figure 6e). The heatmap shows the contribution of each signature to each tumor and Signatures 1, 3, 5, 6, 12, and 20 were the major contributors. Signatures 1 and 5 are clock‐like signatures that are related to aging; signature 3 is seen in tumors with defects in homologous recombination and is related to DSBs; signatures 6 and 20 are DNA mismatch repair signatures, and signature 12 is a liver‐specific signature of unknown etiology that is only observed in HCC. No signatures were observed that are associated with tobacco‐smoking, aflatoxin, or aristocholic acid exposure that are often seen in human HCC.

### 2.7 IGF2 is Overexpressed in a Subset of HCC and Predicts Worse Survival

**Figure 7:**
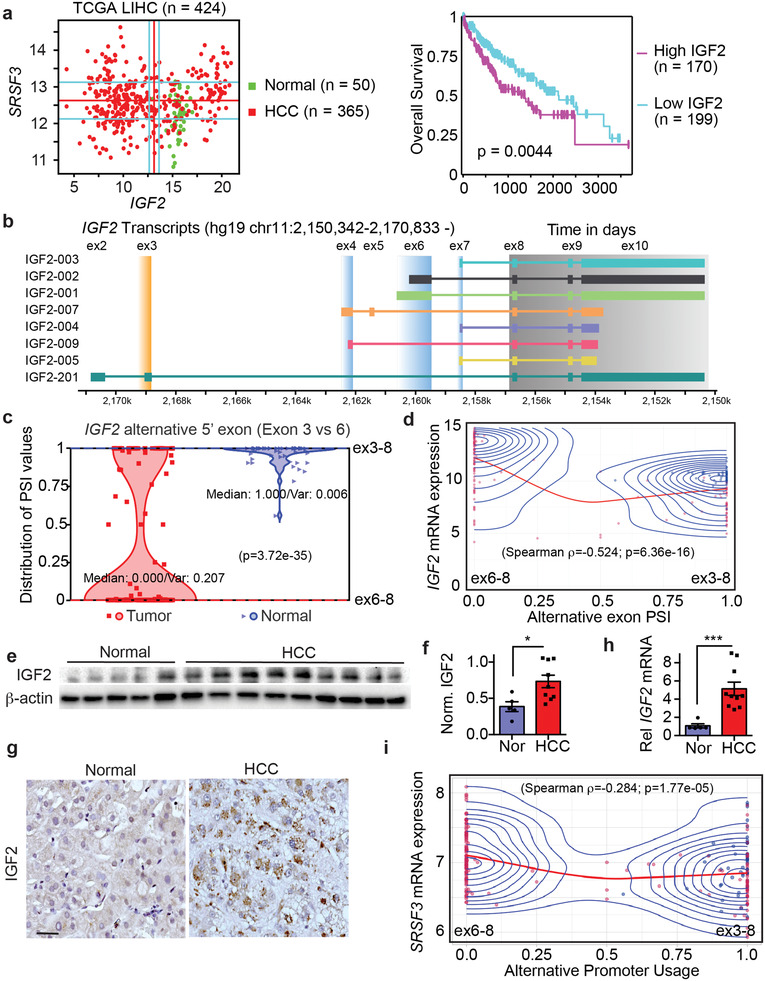
*IGF2* and *SRSF3* mRNAs are over‐expressed in a subset of HCC and predict poor prognosis. a) Scatterplot of *IGF2* and *SRSF3* mRNA expression in the LIHC dataset from TCGA (*n* = 424) and survival plot for patients with *IGF2* mRNA levels above the median versus those with *IGF2* levels below the median. Normal liver samples are colored green; HCC samples are colored red. Kaplan‐Meier survival curve shows that patients with *IGF2* levels above the median (magenta) have a significantly shorter survival than patients with *IGF2* below the median (cyan) (*p* = 0.00044). b) Schematic of transcripts from the *IGF2* gene. Exons 8–10 are the coding exons (gray box) which can be spliced to different upstream 5' UTR exons (blue and orange boxes) that arise from different promoters. Promoter P3 (exon 6) and P4 (exon 7) are fetal promoters, whereas Promoter P0 (exon 3) is the liver specific promoter. c) Violin plot showing alternative splice site usage as percent spliced in (PSI) between exons3–8 versus 6–8. Normal liver samples are shown in blue triangles, HCC in red squares. Median splice site usage and variance is indicated (adj. *p*‐value = 3.72e‐35). d) Correlation between PSI of the fetal P3 promoter (exon 6) versus liver specific P0 promoter (exon 3) and *IGF2* mRNA expression. Individual HCC samples are shown in red, normal samples in blue. Red line is the loess curve fit for the correlation (Spearman rho −0.524, *p*‐value 6.36e‐16), blue lines are the distribution contours. e) Immunoblot of IGF2 protein expression in frozen liver samples from 5 normal liver and 9 NASH‐associated HCC. f) Graph shows quantification of IGF2 levels from the immunoblot. Normal samples are in blue, HCC in red. g) Sections show representative immunohistochemistry for IGF2 in livers from normal and HCC. h) Quantification of *IGF2* mRNA from the same samples by qPCR. i) Correlation between PSI of the P3 and P0 promoter and *SRSF3* mRNA expression (Spearman rho −0.284, *p*‐value 1.77e‐05). Coloring as in panel (d).

It has been reported that IGF2 is overexpressed in HCC.^[^ ^31^ ^]^ We had previously reported that SRSF3 protein expression is lost in HCC, and loss of SRSF3 increases IGF2 expression and predisposes to HCC.^[^ ^3b^ ^]^ To assess whether elevated levels of IGF2 predicted outcomes, we analyzed the TCGA‐LIHC dataset (*n* = 424) to study the relationship between *SRSF3* and *IGF2* expression and patient outcomes. A scatterplot showing the distribution of *SRSF3* and *IGF2* mRNA expression (**Figure** **7**a) revealed that *IGF2* and *SRSF3* mRNA levels do not correlate. *IGF2* showed a greater range in mRNA expression compared to *SRSF3* whose levels were relatively stable consistent with the known autoregulation of its expression.^[^ ^32^ ^]^ ≈16% of HCC samples showed high *IGF2* expression consistent with previous reports.^[^ ^8^ ^]^ To test the effect of high *IGF2* expression on outcomes, we compared survival in the group of patients in the TGCA data with *IGF2* levels above the median value to samples with *IGF2* levels below the median (Figure 7a). Kaplan‐Meier survival plots showed that subjects with high *IGF2* expression had shorter overall survival than those with low levels (*p* = 0.0044).

*IGF2* gene expression is driven from at least 4 different gene promoters and the coding exons 8–10 are alternatively spliced to different upstream 5' exons (Figure 7b). We analyzed the TGCA data for changes in splicing of RNAs from the *IGF2* locus by comparing exon junction reads.^[^ ^33^ ^]^ In the normal liver, all 50 samples had measurable *IGF2* expression, and most samples (45/50) showed predominant (>90%) splicing of exon 3 to exon 8 (Figure 7c) indicating transcription from the distal liver‐specific P0 promoter, with some samples showing a small degree of splicing of both exons 6 and 7 to exon 8 due to use of the proximal fetal promoters P3 and P4 (Figure S8a,b, Supporting Information). We did not observe any exon junctions reads derived from exons 4 or 5 from the P2 promoter in the liver. For the HCC samples, 172 out of 373 had measurable *IGF2* expression and the majority (127/172) showed activation of the fetal promoters P3 and P4. Furthermore, 104 samples showed >90% splicing from exons 6 and 7 to exon 8 indicating a switch to the fetal promoters (Figure 7c and Figure S8a,b, Supporting Information). We also correlated *IGF2* mRNA expression with upstream exon usage. Higher *IGF2* mRNA expression correlated strongly with proximal P3 promoter (exon 6) usage (Figure 7d). As translation of the *IGF2* mRNA is regulated by the IGF2‐mRNA binding proteins (IGFBPs) and miRNAs, we measured IGF2 protein expression in a series of frozen NASH‐associated HCC samples (*n* = 9) and normal liver (*n* = 5) (Table S3, Supporting Information). We did not use HCC adjacent normal tissue as HCC usually occurs in the context of ongoing liver disease and we have published that SRSF3 is lost in early liver disease.^[^ ^4a^ ^]^ The HCC samples showed high levels of IGF2 protein compared to normal liver by immunoblotting (Figure 7e,f) consistent with other published data.^[^ ^9g^ ^]^ Immunohistochemical staining of fixed liver sections confirmed that the IGF2 expression was in hepatocytes (Figure 7g). These results confirm an IHC study using a tissue microarray of 210 HCC specimens, where ≈50% of HCC tumor cells were positive for IGF2 compared to only a few normal hepatocytes.^[^ ^9g^ ^]^ *IGF2* mRNA was also elevated in our HCC samples by qPCR (Figure 7h). As we had previously published increased *Igf2* expression in mice with deletion of *Srsf3* but did not find a correlation of total *IGF2* and *SRSF3* mRNA in the TCGA data (Figure 7a), we examined the correlation of *SRSF3* mRNA with *IGF2* promoter use and found that use of the proximal fetal P3 *IGF2* promoter inversely correlated with the levels of *SRSF3* mRNA (Figure 7i).

### 2.8 SRSF3 Levels Predicts HCC Survival

**Figure 8:**
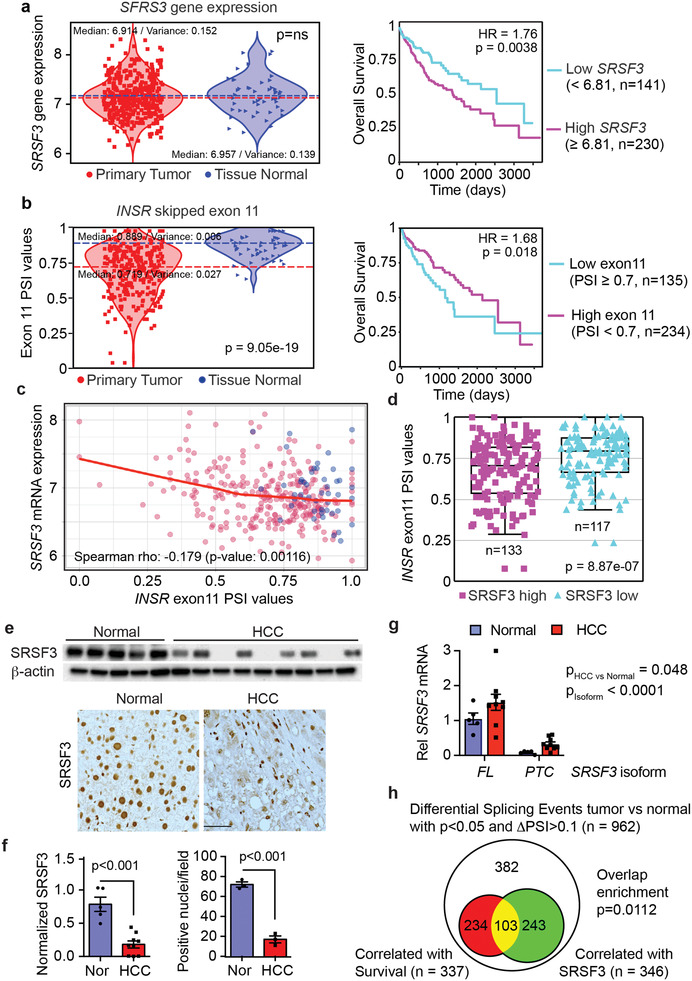
Alterations in SRSF3‐dependent splicing predict poor prognosis. a) Violin plot showing *SRSF3* mRNA levels in the TCGA‐LIHC dataset on left. Normal liver samples are shown in blue triangles, HCC in red squares. Median splice site usage and variance are indicated. Survival curve for HCC patients with greater than (magenta, *n* = 230) versus less than (cyan, *n* = 141) median *SRSF3* expression in the LIHC dataset (HR = 1.76, *p* = 0.0038) on right. b) Violin plot showing skipping or inclusion of exon 11 in the INSR gene on left. Plot shows PSI—percent spliced in values, that is, exon inclusion, for normal (blue) and HCC (red). Statistical significance for difference in PSI values (*p* = 9.05e‐19). Survival plot for patients with INSR exon 11 PSI < 0.7 (cyan) versus > = 0.7 (magenta) on right (HR = 1.68, *p* = 0.018). c) Correlation of *SRSF3* mRNA expression with *INSR* exon 11 inclusion (Spearman rho −0.179, *p*‐value 0.00116). Tumor samples are shown in red and normal in blue. Red line is the loess curve fit for the correlation. d) *INSR* exon 11 inclusion in HCC samples stratified by *SRSF3* mRNA expression. The SRSF3 high group comprises samples with *SRSF3* expression above the median value (*n* = 133), whereas the SRSF3 low group contains samples with *SRSF3* expression below the median value (*n* = 117). The boxplot shows median value, interquartile range, and 95% confidence interval. e) Immunoblotting normal and HCC extracts for SRSF3 protein and immunohistochemistry for SRSF3 on normal and HCC liver sections. f) Graphs show quantification of immunoblot and immunohistochemistry. g) Quantification of *SRSF3* transcript isoforms in normal and HCC samples by qPCR. *FL* indicates transcript *SRSF3‐202* encoding full length SRSF3. *PTC* indicates transcript *SRSF3‐203* including exon 4 and encoding a premature stop codon. Normal samples are in blue, HCC in red. 2‐way ANOVA indicates a significant increase in HCC (*p* = 0.048) and a significant difference in isoform expression (*p* < 0.0001) but no interaction. h) Venn diagram showing overlap of differential splicing events (ΔPSI>0.1) associated with overall survival versus those correlated with *SRSF3* expression in the LIHC dataset. For bar graphs, bars show mean ± SEM. Statistical significance by Welch's two sample *t*‐test, 2‐way ANOVA, logistic regression, or Χ^2^ test as appropriate.

*SRSF3* mRNA expression did not vary as greatly as *IGF2* in the TCGA‐LIHC dataset (Figure 7a). As *SRSF3* mRNA levels did not separate into distinct groups (**Figure** **8**a), we split the HCC samples based on expression above and below the median for a survival analysis (Figure 8a). SRSF3 negatively regulates its own expression, so high *SRSF3* mRNA would indicate low SRSF3 splicing activity.^[^ ^32^ ^]^ Survival in the high *SRSF3* mRNA expressing group was significantly worse than the low expressing group (*p* = 0.0038). Consequently, we analyzed known SRSF3‐dependent splicing events, *INSR* exon 11, *FN1* exon 33, *MYO1B* exon 23, and *SLK* exon 13, in the TCGA dataset.^[^ ^3^, ^4^ ^]^ All four splicing events were altered in the HCC samples (Figure 8b and Figure S8c–e, Supporting Information). The two SRSF3‐promoted exons (*INSR* and *SLK*) were decreased (*p* = 9.05e‐19 and 1.13e‐19), whereas the two SRSF3‐inhibited exons (*FN1* and *MYO1B*) were increased in HCC (*p* = 2.7e‐64 and 1.23e‐6). Tumor‐associated splicing events correlated with worse survival (hazard ratios 1.5 to 2.17, *p*‐values 2.54e‐4 to 0.0349) implying that SRSF3‐dependent splicing activity is associated with better survival (Figure 8b and Figure S8c–e, Supporting Information).

We also confirmed that inclusion of *INSR* exon 11 negatively correlated (*p* = 0.00116) with *SRSF3* mRNA levels (Figure 8c) and that exon 11 inclusion was greater in the patient samples with low *SRSF3* mRNA (Figure 8d). To confirm that SRSF3 protein is lost in HCC, we assessed SRSF3 expression in our NASH‐associated HCC by immunoblotting. The HCC samples showed lower SRSF3 protein expression compared to normal liver by western blot and immunohistochemical staining of fixed liver sections. The majority of hepatocyte nuclei in HCC stained weakly for SRSF3 while normal liver hepatocyte nuclei stained strongly (Figure 8e,f). SRSF3 staining was still evident, however, in Kupffer cells, stellate cells, and immune cells in the tumors. The level of SRSF3 and IGF2 protein quantified by western blot were negatively correlated (*r* ^2^ = 0.387, *p* = 0.0175) in these HCC samples (Figure S8f, Supporting Information). Autoregulation of SRSF3 expression is accomplished by SRSF3‐dependent changes in the ratio of the transcripts for the full‐length isoform (FL) and the truncated isoform (PTC) that is subject to non‐sense mediated decay. Therefore, we measured expression of the major (*SRSF3‐FL*) and minor *SRSF3* transcripts (*SRSF3‐PTC*) by qRT‐PCR in RNA from our frozen samples. Both *SRSF3* FL and PTC transcripts were increased in HCC samples (Figure 8f).

We then took an unbiased approach and analyzed alternative splicing events in the TGCA LIHC dataset. Using principal component analysis, the normal samples were clustered together and showed separation from the tumor samples for both gene expression and splicing events (Figure S9a, Supporting Information). For gene expression the major contributors to the PCA separation were cytochrome P450 genes involved in drug metabolism (CYPs) and detoxification (GLYAT) whereas the splicing events contributing to the PCA separation were involved in growth‐factor signaling and cell proliferation/apoptosis (Figure S9b and Tables S4,S5, Supporting Information). We then analyzed the changes in alternative splicing between the tumor and normal tissue. After FDR correction, 5,623 splicing events (SEs) were significantly altered compared to normal liver in the TCGA data out of annotated 34,396 splicing events that were derived from 249,576 splice junctions (Figure S9c, Supporting Information). These were then filtered to include only those splicing events with ΔPSI >0.1, leaving 962 significant SEs (Figure 8g, Table S6, Supporting Information). We then analyzed which of these events correlated with *SRSF3* expression or with overall survival. Of the 962 significant SEs with ΔPSI >0.1, 346 correlated with *SRSF3* expression, and 337 correlated with survival (Figure 8g). There was a significant overlap of SEs that correlated with both *SRSF3* expression and survival (*p* < 0.0112) suggesting that in general SRSF3‐dependent splicing is associated with better survival as we had shown for *INSR, SLK*, and *MYO1B*. Similar correlations and enrichments were found in the larger dataset of 5,623 SEs (Figure S9c, Supporting Information). The top 103 genes with ΔPSI>0.1 that correlated with both survival and SRSF3 expression (Figure 8c) were functionally mapped to pathways and protein interaction networks to understand the role of the affected proteins. This analysis revealed enrichment for SEs in genes involved in proliferation/mitosis, chemotaxis, and signaling (Figure S9d and Table S7, Supporting Information) and a significant protein interaction network with many members implicated in tyrosine kinase/PI‐3Kinase signaling and mRNA splicing (Figure S9e, Supporting Information). The SRSF3 correlation plots for the top 20 SEs associated with survival are shown in Figure S10, Supporting Information.

## 3 Discussion

We had previously reported that loss of SRSF3 induces IGF2 expression and predisposes to spontaneous HCC with aging.^[^ ^3b^ ^]^ Expression of IGF2 is seen at 1 month and is further increased in liver and tumors that arise after 12–15 months. In this study we investigated the role of IGF2 in this liver phenotype and showed that loss of IGF2 reduced liver inflammation and fibrosis, but not steatosis, in mice lacking SRSF3 in hepatocytes. We showed that loss of SRSF3 causes DNA damage and activation of the p53 and ATM DNA‐damage response pathways. Tumors from SKO mice not only showed mutational signatures of defective homologous recombination consistent with the observed DSBs, but also showed signatures of defective DNA mismatch repair that is consistent with the decreased expression of DNA repair enzymes. The tumors also showed a very specific human HCC signature that is not seen in other cancers. The surprising observation was that co‐deletion of IGF2 in the SRSF3‐KO hepatocytes prevented tumors and reduced hepatocyte apoptosis and proliferation, DNA damage, p53, and ATM activation. Most of the data suggesting a role for IGF2 in HCC are correlative, and this is the first report that IGF2 can directly cause HCC. IGF2 is deleted only in the hepatocyte and circulating levels are not significantly different from the control Flox mice indicating that this is an autocrine rather than an endocrine effect of circulating IGF2.

The role of SRSF3 in preventing DNA damage is supported by several publications. Knockdown of SRSF3 in A2780 ovarian cancer cells decreases expression of BRCA1, BRIP1, and RAD51, increases *γ*‐H2A.X levels, and impairs homologous recombination of a GFP reporter gene.^[^ ^34^ ^]^ SRSF1 and SRSF3 inhibit RNA:DNA R‐loop formation at sites of active transcription and prevent double‐strand DNA breaks.^[^ ^35^ ^]^ The splicing factor SLU7 also prevents R‐loop formation and DNA damage by modulating SRSF3 splicing and expression.^[^ ^36^ ^]^ Our results are consistent in that loss of SRSF3 caused double‐strand breaks, and we now extend those findings to show that induction of IGF2 following loss of SRSF3 is important for induction of DNA damage. Normally the liver is not responsive to IGF2 as hepatocytes do not express the IGF1R, but loss of SRSF3 causes skipping of exon 11 of the INSR allowing IGF2 to activate insulin signaling via INSR‐A.^[^ ^3^, ^18^ ^]^ The importance of this mechanism is supported by the observation that loss of the related splicing factor SRSF1 did not alter INSR splicing and did not cause HCC despite elevating IGF2. This SRSF3:IGF2:INSR‐A axis may be relevant to human disease as expression of INSR‐A is increased in 75–80% of HCC samples and is associated with stemness markers and shorter patient survival.^[^ ^37^ ^]^ In contrast, the INSR isoform including exon 11 (INSR‐B) is associated with increased differentiation and decreased stemness and proliferation.^[^ ^38^ ^]^ The failure of the anti‐IGF1R mAb cixutumumab in a phase II trial supports the proposition that IGF2 signaling through the INSR‐A may be more important in HCC.^[^ ^39^ ^]^ Insulin can also signal via INSR‐A but it causes receptor downregulation and inhibition of insulin signaling, which IGF2 does not.^[^ ^40^ ^]^

The role of IGF2 in driving hepatocyte proliferation is supported by previous studies. Pericentral hepatocytes produce IGF2 to trigger regeneration in response to liver injury ^[^ ^41^ ^]^ and IGF2 is key mitogen driving repopulation of the liver in a mouse model of hereditary tyrosinemia.^[^ ^42^ ^]^ The ability of IGF2 overexpression to cause DNA damage, however, is less understood. IGF2 and insulin signaling is suppressed by DNA damage as wild‐type p53, but not mutant p53, inhibits both the IGF1R and INSR gene promoters ^[^ ^43^ ^]^ and p53 also represses IGF2 expression.^[^ ^44^ ^]^ The regulation is likely reciprocal as cancer‐cell secreted IGF2 represses p53 in fibroblasts potentially via AKT phosphorylation of MDM2.^[^ ^45^ ^]^ IGF2 is an important mitogen in p53 mutant tumors as conditional deletion of *Igf2* reduces tumors in *p53* null mice.^[^ ^46^ ^]^ Here we show that loss of SRSF3 caused DNA damage and activation of p53 signaling in an IGF2‐dependent manner. This could be a direct effect as we also showed that IGF2 suppresses DNA‐damage response pathways and caused DNA damage in HepG2 cells. In support of the ability of IGF2 to cause such damage, YAP1 promotes genomic instability in medulloblastoma by inducing IGF2, activating AKT, and inhibiting ATM, thus allowing cells with un‐repaired DNA to enter mitosis,^[^ ^47^ ^]^ and IGF2 reduces p53. Our results also uncovered a potential SRSF3‐IGF2‐SRSF3 feedforward loop. Loss of SRSF3 increases IGF2 expression which in turn represses SRSF3 expression. This type of loop may exacerbate liver damage and progression to HCC once the initial metabolic insult causes SRSF3 degradation and may hinder the reversal of NASH. Consistent with this idea, expression of a degradation resistant mutant of SRSF3 prevents and reverses hepatitis and fibrosis in response to a NASH diet.^[^ ^4a^ ^]^

Human data also support the proposition that IGF2 overexpression and SRSF3 loss are important drivers of HCC. As mentioned earlier, elevated *IGF2* mRNA expression correlated with shorter survival in HCC, was associated with a switch from normal liver‐specific promoter P1 to the fetal P3 promoter in the *IGF2* gene, and was inversely correlated with *SRSF3* mRNA levels. As these fetal IGF2 transcripts are targets for translation stimulation by the IGF2BPs, this may explain the higher IGF2 protein levels that are found in HCC. We found that SRSF3 levels were reduced in NASH‐associated HCC, which agrees with other studies reporting loss or functional inhibition of SRSF3 in HCC,^[^ ^4^, ^5^, ^6^ ^]^ and inversely correlated with IGF2 levels. Furthermore, we found that alteration in known SRSF3‐dependent splicing events correlated with worse survival, and that an unbiased analysis of splicing events associated with better survival in the LIHC cohort showed an enrichment for splicing events that correlated with SRSF3 expression.

## 4 Conclusion

In conclusion, our findings support the conclusion that hepatic IGF2 expression is a carcinogenic driver in a mouse model of aging‐related HCC by causing DNA damage and supporting hepatocyte proliferation that would allow the accumulation of somatic mutations. Our findings also support the importance of loss of SRSF3 in triggering IGF2 expression and altering INSR splicing to allow IGF2 signaling via IR‐A in hepatocytes. Therapies specifically targeting SRSF3 and/or IGF2 may prove useful in a subset of HCC with reduced SRSF3 function and IGF2 overexpression.

## 5 Experimental Section

### Human Tissue Samples

Human HCC and normal tissue samples were obtained from the University of Minnesota Liver Tissue and Cell Distribution Service (UM‐LTCDS), and the Department of Pathology at the VASDHS. Subject characteristics are provided in Table S3, Supporting Information. Liver histology and RNA extraction were performed as described previously.

### HCC Mouse Model

All protocols involving animals were approved by the Institutional Animal Use and Care Committee of UCSD (S06319). Mouse procedures conformed to the Guide for Care and Use of Laboratory Animals of the US National Institutes of Health. C57BL/6J mice were purchased from Jackson Labs (Bar Harbor, ME). Hepatocyte‐specific SRSF3‐KO (SKO) mice were generated as described previously.^[^ ^3a^ ^]^ *Igf2* floxed mouse containing loxP sites on either side of exons 4–6 were obtained from Dr. Miguel Costancia, University of Cambridge.^[^ ^48^ ^]^ A hepatocyte‐specific double deletion of SRSF3 and IGF2 (DKO), or single IGF2 deletion (IGKO) mice was generated by breeding *Igf2^fl:fl^* mice with the SKO (*Srsf3^fl/fl^:Alb‐cre*) mice to generate DKO (*Igf2^fl/fl^:Srsf3^fl/fl^:Alb‐cre*) and IGKO (*Igf2^fl/fl^:Alb‐cre*) mice. Hepatocyte‐specific SRSF1 knockout mice were generated by crossing *Srsf1*‐floxed mice to the *Alb‐Cre* mice as described for the *Srsf3* mice.^[^ ^3a^ ^]^

### Glucoregulatory Assessments

For the glucose tolerance test (GTT), 8–10 mice per group were fasted for 6 h prior to GTT. Blood glucose was measured by tail bleed at 0 min and then 1 g kg^−1^ glucose injected intraperitoneally. Blood glucose was monitored at intervals up to 120 min using a glucose meter (Easy Step Blood Glucose Monitoring System, Home Aide Diagnostics, Inc., Deerfield Beach, FL). For the insulin tolerance test (ITT) mice were fasted for 6 h and blood glucose measured at 0 min prior to injection of 0.65 U kg^−1^ insulin intraperitoneally and then at intervals up to 120 min. Terminal fasting glucose was also measured following 6 h fasting. Terminal fasting plasma insulin, IGF1, IGF2, and GH were measured by electro‐chemiluminescense assay (Meso Scale Diagnostics, Rockville, MD).

### Cell Culture and Treatment

Primary hepatocytes were obtained from 1 month old mice of flox, SKO, DKO, and IGKO and primary hepatocyte cells were isolated as described previously.^[^ ^4a^ ^]^ HepG2 cells were obtained from ATCC and maintained in 1× DMEM supplemented with 10% fetal bovine serum, and 100 U mL^−1^ of penicillin and 100 µg mL^−1^ of streptomycin at 37 °C in an atmosphere of 5% CO2. HepG2 cells were starved for 8–12 h before stimulation with IGF2. The PI3K inhibitor wortmannin (50 nm) was added 30 min before stimulation. Starved cells were stimulated with 100 ng mL^−1^ IGF2 for 30 min, then IGF2 was removed, and cells were maintained in serum‐free media for 24 and 48 h with or without wortmannin.

### Gene Expression

Total RNA was extracted from the cells and tissues using RNA‐Stat60 (Tel‐Test Inc. Friendswood, TX) following the manufacturer's instructions. First‐strand cDNA was synthesized using a High‐Capacity cDNA synthesis kit (Applied Biosystems, Waltham, MA). Quantitative PCR was performed on MJ Research Chromo4 or Bio‐Rad CFX96 instruments (Bio‐Rad, Hercules, CA). Gene expression levels were calculated after normalization to the housekeeping gene, m36B4, and GAPDH using the 2‐∆∆Ct method and expressed as relative mRNA levels compared to the control. Primers are listed in Table S8, Supporting Information.

### RNAseq Analysis

LIHC gene counts, exon‐junction counts, and metadata files were downloaded from the TCGA website. Data was loaded into the Psichomics program (Saraiva‐Agostinho Lab, University of Lisbon, Portugal), gene counts were normalized using trimmed mean of M‐values (TMM), log‐transformed, and IDs converted to gene symbols. Exon‐junction counts were converted to the percent‐spliced in metric (PSI) and annotated using human hg19/GRCh37. Individual gene expression or alternative splicing events were visualized on violin plots. Survival analysis was performed using selected gene expression or PSI cutoffs, then Kaplan‐Meier curves plotted and Cox proportional hazard models were performed. Correlation analysis between gene expression and an alternative splicing event was performed using Spearman rank correlation. Expression data for INSR and IGF1R in normal human liver was downloaded from the HPA and GTEX databases. Enrichment analysis and protein‐interaction network generation of the genes altered in the SRSF3 knockout hepatocytes were performed using Metascape (Benner Lab, UCSD).

### Exome Sequencing

Genomic DNA was isolated from tumors and normal liver tissue from the SKO mice. Exome enrichment was performed using the SureSelectXT Mouse all exon kit (Agilent Technologies, Santa Clara, CA) then sequenced by paired‐end 150 nucleotide sequencing (Illumina, San Diego, CA). Raw reads were cleaned and aligned to the mouse mm9 genome using the Burrows‐Wheeler Aligner. Variants were called with GATK and somatic mutations and indels were analyzed by MuTect2 and Strelka, and functional effects were assessed using Variant Effect Predictor (VEP) using SIFT and Polyphen‐2. Mutational signatures were analyzed with MutationalPatterns, SomaticSignatures, and YAPSA.

### Immunoblot Analysis

Cells lysates were isolated as previously described.^[^ ^4a^ ^]^ Equal amounts of cellular protein (10 µg) were separated by SDS‐PAGE using 4−15% or 20% Criterion precast polyacrylamide gels (Bio‐Rad), transferred to PVDF membranes (MilliporeSigma, Burlington, MA), blocked with 5% BSA for 1 h at RT and immunoblotted with primary antibodies overnight at 4 °C followed by HRP‐conjugated secondary antibodies at room temperature for 1 h, washed 3× in TBS‐Tween20 then developed using a chemiluminescent substrate kit (Pierce, Rockford, IL). Antibodies used for immunoblotting were mouse monoclonal 7B4 anti‐SRSF3 antibody (1:1000 dilution, ATCC CRL‐2384, Manassas, VA), anti‐IGF2 rabbit monoclonal antibody (1:1000 dilution, ab6328, Abcam, Cambridge, MA), anti‐*γ*‐H2A.X rabbit (1:1000 dilution, Cell Signaling, 2577), anti‐p‐P53 Ser15 rabbit (1:1000 dilution, Cell Signaling, 82530) anti‐p‐P53 Ser37 rabbit (1:1000 dilution, Cell Signaling, 9289), anti‐p‐P53 Ser392 rabbit (1:1000 dilution, Cell Signaling, 9281), anti‐P53 mouse (1:200 dilution, Santa Cruz Biotechnology Inc, sc‐126), anti‐P21 mouse (1:200 dilution, Santa Cruz Biotechnology Inc, sc‐166630), anti‐MDM2 mouse (1:200 dilution, Santa Cruz Biotechnology Inc, sc‐965), anti‐MDC1 rabbit (1:1000 dilution, Millipore Sigma, PL0016), anti‐p‐ATM Ser1981 rabbit (1:1000 dilution, Cell Signaling, 13050), anti‐ATM rabbit (1:1000 dilution, Cell Signaling, 2873), anti‐XRCC1 rabbit (1:1000 dilution, Cell Signaling, 2735), anti‐MSH2 rabbit (1:1000 dilution, Cell Signaling, 2017), anti‐XPD rabbit (1:1000 dilution, Cell Signaling, 11963), anti‐phospho‐Ser473‐AKT rabbit (1:1000 dilution, Cell Signaling, 4060), anti‐AKT, rabbit (1:1000 dilution, Cell Signaling, 4685), anti‐phospho‐Tyr1158,1162,1163‐INSR rabbit (1:1000 dilution, Bio source International, CA, USA), anti‐phospho‐Thr202/Tyr204‐ERK, rabbit (1:1000 dilution, Cell Signaling, 4370), anti‐ERK1 rabbit (1:1000 dilution, Cell Signaling, 4695). HRP labeled anti‐mouse (sc516102) or anti‐rabbit (sc2357) secondary antibody (1:5000 dilution, Santa Cruz Biotechnology, Santa Cruz, CA). Blots were quantified using a Gel‐Doc imaging system (Bio‐Rad).

### Immunohistochemistry

Immunohistochemistry was performed on formalin‐fixed, paraffin‐embedded human and mouse liver sections. Tissues’ staining was performed as described previously.^[^ ^4a^ ^]^ Tissue slides were incubated overnight at 4 °C with mouse anti‐SRSF3 (7B4, 1:100 dilution), rabbit anti‐IGF2 rabbit monoclonal antibody (1:100 dilution, ab6328, Abcam, Cambridge, MA), rabbit anti‐Ki67 (1:2000 dilution, ab15580, Abcam, Cambridge, MA), rabbit anti‐cleaved caspase 3 (1:1000 dilution, Cell Signaling, 9664), or rabbit anti‐*γ*‐H2A.X, rabbit (1:100 dilution, Cell Signaling, 2577) antibodies in blocking buffer. Slides were visualized using a VectorSain ABC immunohistology kit (Vector Labs, Burlingame, CA).

### Statistical Analysis

Data were analyzed by 1‐way or 2‐way ANOVA followed by Tukey multiple comparison post‐test, or Students’ *t*‐test as appropriate using Prism (Graph Pad, La Jolla, CA) or in R statistical software (v3.4.4). Normality was assessed by D'Agostino‐Pearson omnibus normality test. Results were expressed as Mean +/−Standard Error and considered significant with *p* < 0.05. Linear regression and Chi‐squared contingency table analysis were performed using Prism.

## Conflict of Interest

The authors declare no conflict of interest.

## Supporting information

Supporting Information

## Data Availability

The data that support the findings of this study are available in the supplementary material of this article.
