# Supplementary material for: Hepatocyte Deletion of IGF2 Prevents DNA Damage and Tumor Formation in Hepatocellular Carcinoma
Source: Adv Sci (Weinh). 2022 May 26;9(21):2105120. doi: 10.1002/advs.202105120 (PMC9313545; doi:10.1002/advs.202105120)
Supplement: Supplementary file 1 — Supporting Information [file ADVS-9-2105120-s001.pdf]

ADVANCED SCIENCE
Open Access

Supporting Information

for *Adv. Sci.*, DOI 10.1002/advs.202105120

Hepatocyte Deletion of IGF2 Prevents DNA Damage and Tumor Formation in Hepatocellular Carcinoma

*Deepak Kumar, Manasi Das, Alexis Oberg, Debashis Sahoo, Panyisha Wu, Consuelo Sauceda, Lily Jih, Lesley G. Ellies, Magda T. Langiewicz, Supriya Sen and Nicholas J. G. Webster**

Hepatocyte Deletion of IGF2 Prevents DNA Damage and Tumor Formation in Hepatocellular Carcinoma

*Deepak Kumar, Manasi Das, Alexis Oberg, Debashis Sahoo, Panyisha Wu, Consuelo Sauceda, Lily Jih, Lesley G Ellies, Magda T. Langiewicz, Supriya Sen and Nicholas J.G. Webster**

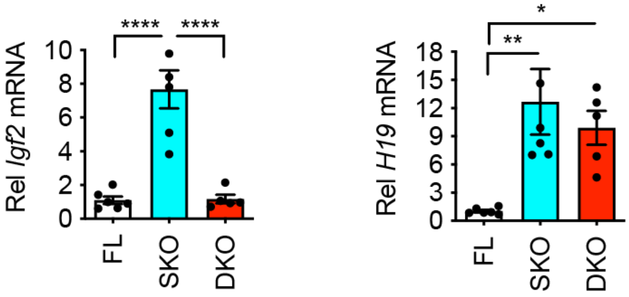

Figure S1: Genetic deletion of *Igf2* in hepatocytes reduces *Igf2* expression but not *H19*. *Igf2* and *H19* expression were measured in liver by qPCR. For all graphs, asterisks show statistical significance by ANOVA; *p<0.05, **p<0.01, and ****p<0.0001 for the indicated comparison. Individual data points are shown (n = 5 or 6 per group). Flox mice are shown in white, SKO mice in blue, and DKO mice in red.

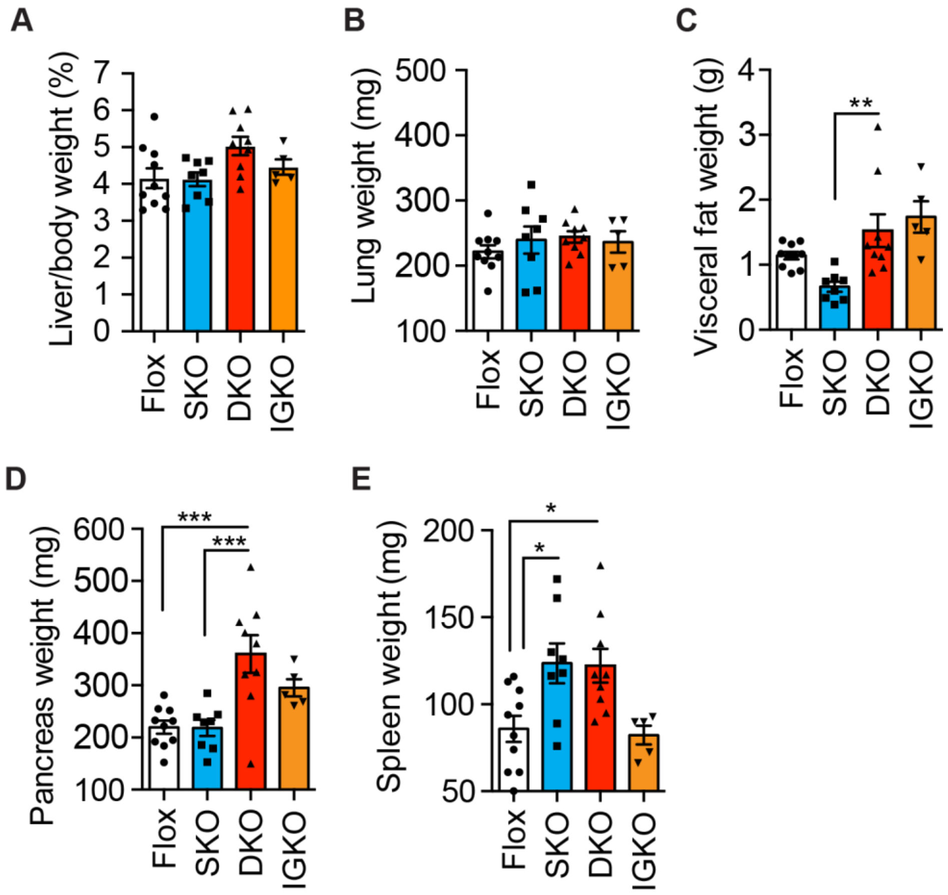

Figure S2: Effect of genetic deletion of *Igf2* in hepatocytes on tissue weights. A: Liver to body weight ratio. **B:** Lung weight. **C:** Visceral fat depot weight. **D:** Pancreas weight. **E:** Spleen weight. For all graphs, asterisks show statistical significance by ANOVA; *p<0.05, **p<0.01, and ****p<0.0001 for the indicated comparison. Individual data points are shown (n = 5 to 10 per group). Flox mice are shown in white, SKO mice in blue, DKO mice in red, and IGKO mice in orange.

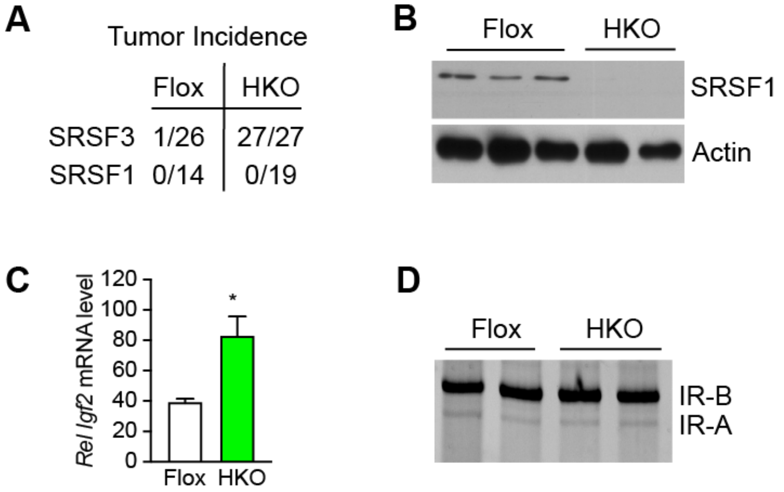

Figure S3: Hepatocyte-specific knockout of SRSF1 does not cause HCC at 12-18 months. A: Tumor incidence in SRSF3-HKO and SRSF1-HKO mice compared to their Flox control littermates. Data for SRSF3-HKO taken from ref 13. Mice were sacrificed between 12 and 18 months of age. Ratio is the number of mice with tumors/number of mice. **B:** Expression of SRSF1 in primary hepatocytes from mice at 5 weeks of age. SRSF1 was measured by immunoblotting. β-actin was used as a loading control. **C:** *Igf2* mRNA levels in SRSF1-HKO mice versus Flox littermates (n=3/group). Asterisk shows statistical significance by 2-sided t-test; *p<0.05. **D:** Insr exon 11 splicing by RT-PCR in RNA extracted from livers of SRSF1-HKO and Flox mice. Upper band shows *Insr exon11+* (IR-B) and lower band is *Insr exon11-* (IR-A).

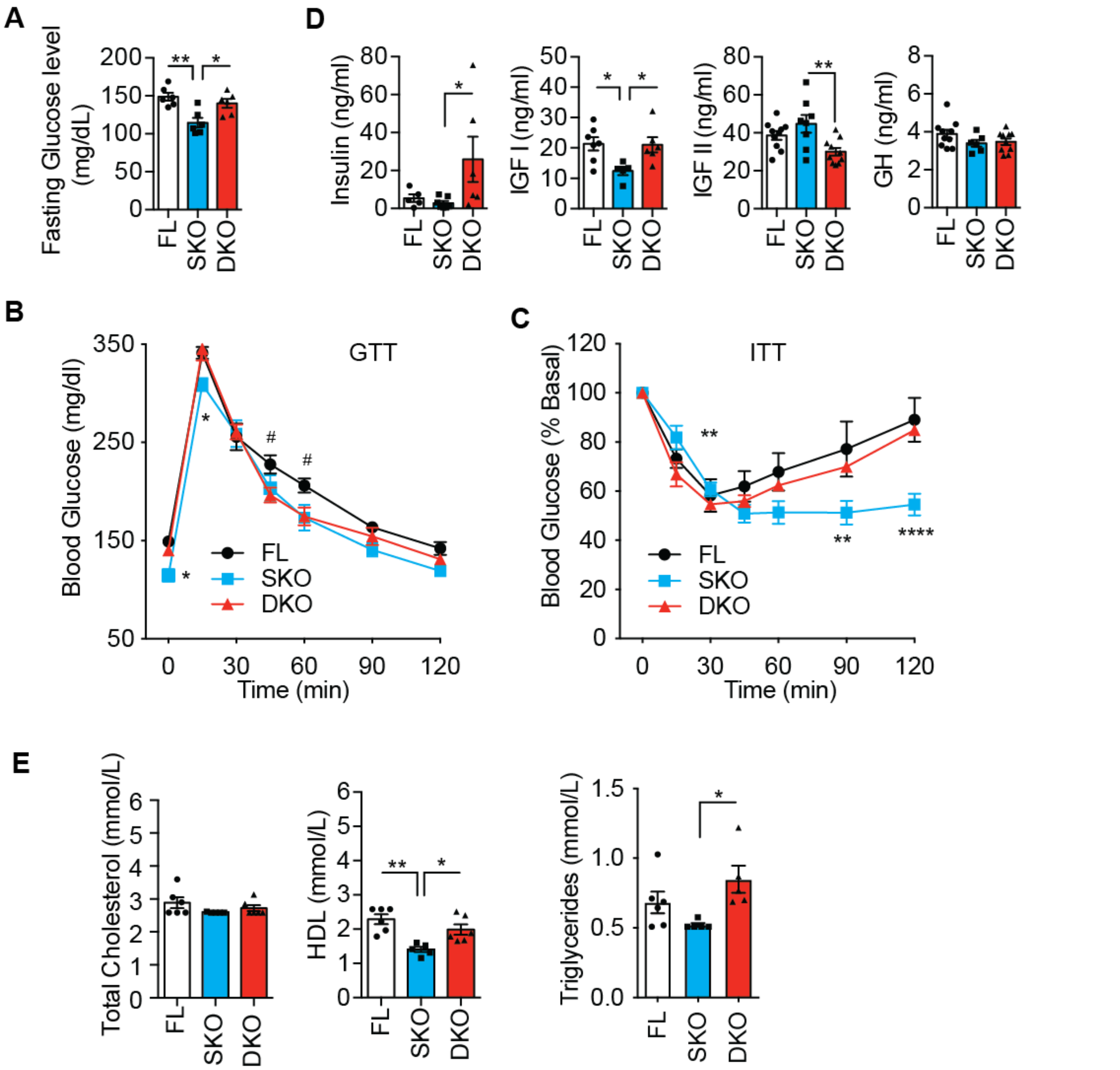

Figure S4: Metabolic effect of genetic deletion of *Igf2* in hepatocytes. A: Fasting blood glucose at sacrifice. Flox mice are shown in white, SKO mice in blue, and DKO mice in red, n=5-6 per group. **B:** Intraperitoneal glucose tolerance tests (n = 6/group). Fasted mice were given a bolus of 1 g/kg glucose by ip injection and blood glucose levels monitored over 2 h. Repeated measures 2-way ANOVA indicates a significant time effect ($p<0.0001$), genotype effect ($p=0.0325$, and interaction ($p=0.0324$). Asterisks indicate statistical significance SKO vs DKO or Flox. Hashtags indicate significance Flox vs SKO or DKO. **C:** Intraperitoneal insulin tolerance tests (n= 6/group). Fasted mice were given a bolus of 0.65 U/kg insulin by ip injection and blood glucose levels monitored over 2 h. Repeated measures 2-way ANOVA indicates a significant time effect ($p<0.0001$), and genotype-time interaction ($p<0.0001$) but no genotype effect (ns). Asterisks indicate statistical significance SKO vs DKO or Flox. **D:** Fasting insulin, IGF1, IGF2 and growth hormone levels after sacrifice (n = 8 to 10 per group). **E:** Fasting total cholesterol, HDL cholesterol and triglycerides (n = 5 to 6 per group). For all bar graphs, asterisks show statistical significance by ANOVA; *$p<0.05$ and **$p<0.01$, for the indicated comparison. Individual data points are shown.

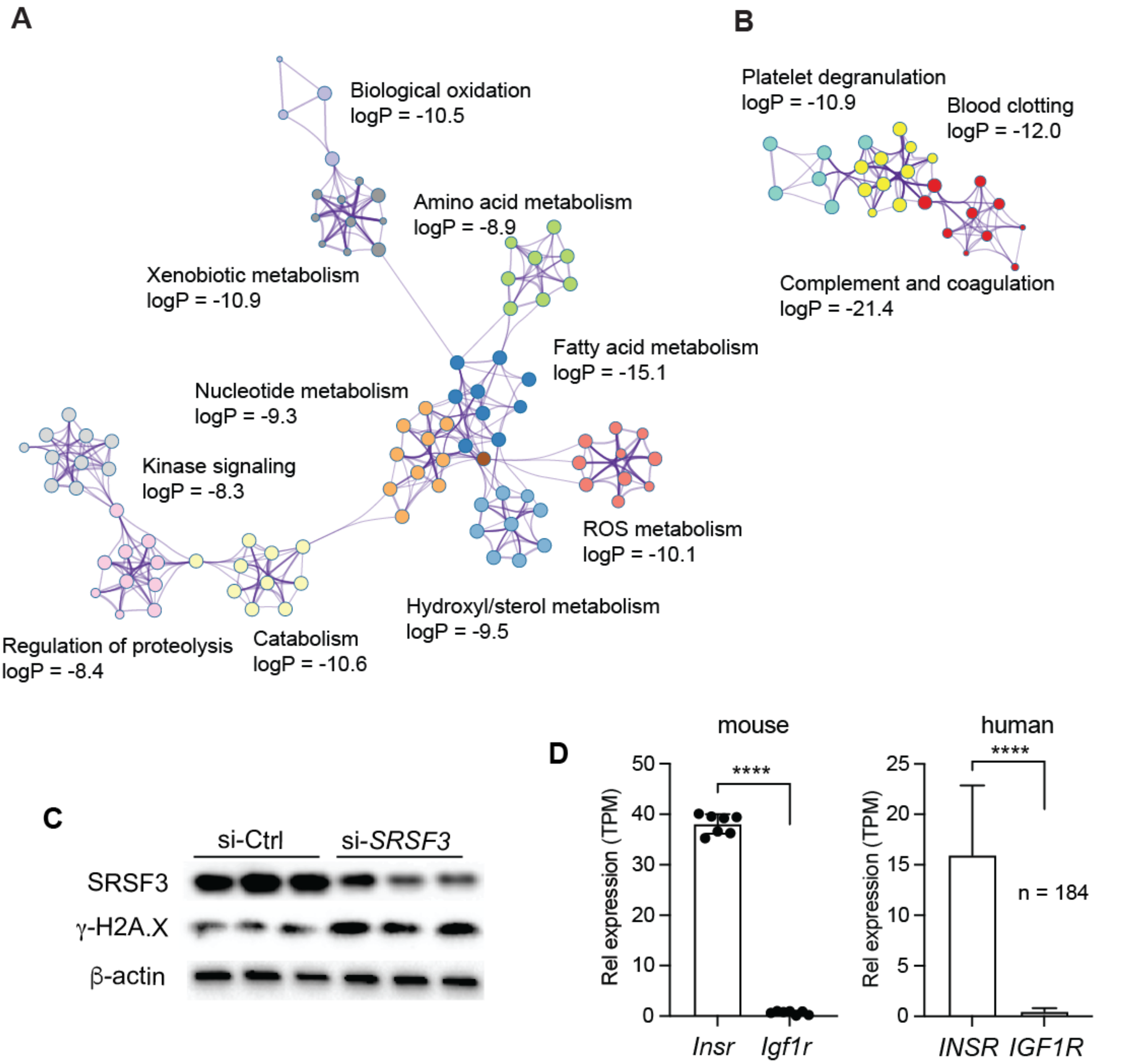

Figure S5: Genes altered in SRSF3 knockout hepatocytes are enriched for genes involved in metabolism and blood hemostasis. A: Cluster of metabolic terms and pathways that are enriched for genes altered in SRSF3 knockout hepatocytes using Metascape. **B:** Cluster of hemostasis terms and pathways that are enriched for genes altered in SRSF3 knockout hepatocytes. The enrichment scores for each sub-cluster are given. **C:** Knockdown of SRSF3 *in vitro* in HepG2 cells increases γ-H2A.X levels, which is a marker of double stranded DNA breaks. Top panel shows knockdown of SRSF3, middle panel shows γ-H2A.X, and bottom panel shows β-actin loading control. **D:** *Insr* and *Igf1r* expression in mouse liver (n=7/group) and in human liver (n=184/group). Human data are analyzed in the HPA and GTex databases. For bar graphs, asterisks show statistical significance by 2-sided t-test; ****p<0.0001.

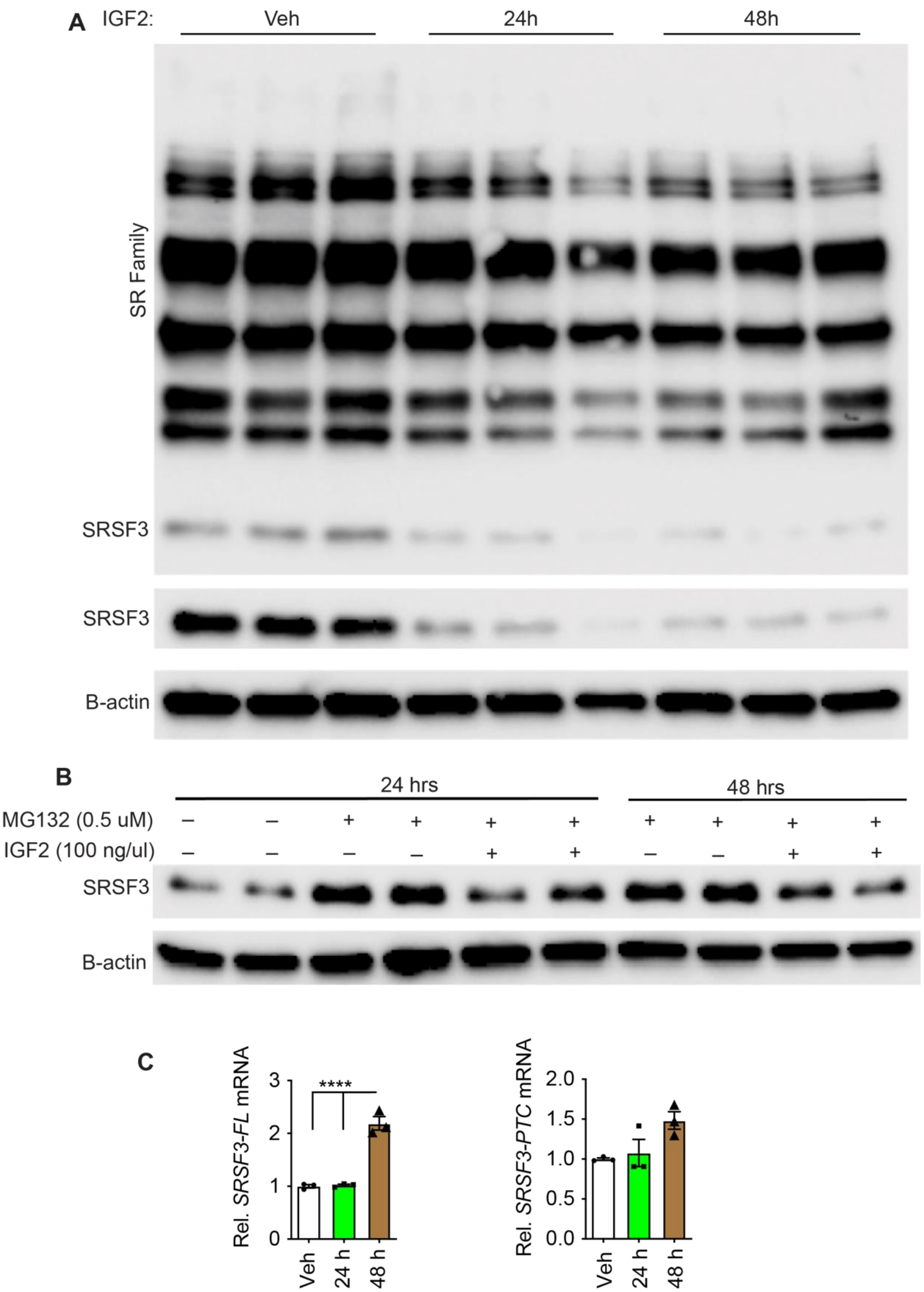

Figure S6: IGF2 treatment reduces SRSF3 in HepG2 cells. A: HepG2 cells treated with 100 ng/nl IGF2 for 24 and 48 h. Extracts were immunoblotted with an antibody for phosphorylated SR proteins (mAb104). Extracts were also blotted for SRSF3 and for β-actin as a loading control. **B:** HepG2 cells were pretreated with the proteosomal inhibitor MG132 for 30 min then stimulated with IGF2 for 24 and 48 h. SRSF3 was immunoblotted on cell extracts. **C:** Srsf3-FL and PTC transcript levels after IGF2 stimulation by qPCR. Data are shown as mean±SEM. Asterisks show statistical significance by ANOVA; *p<0.0001 for the indicated comparison. Individual data points are shown (n=3).

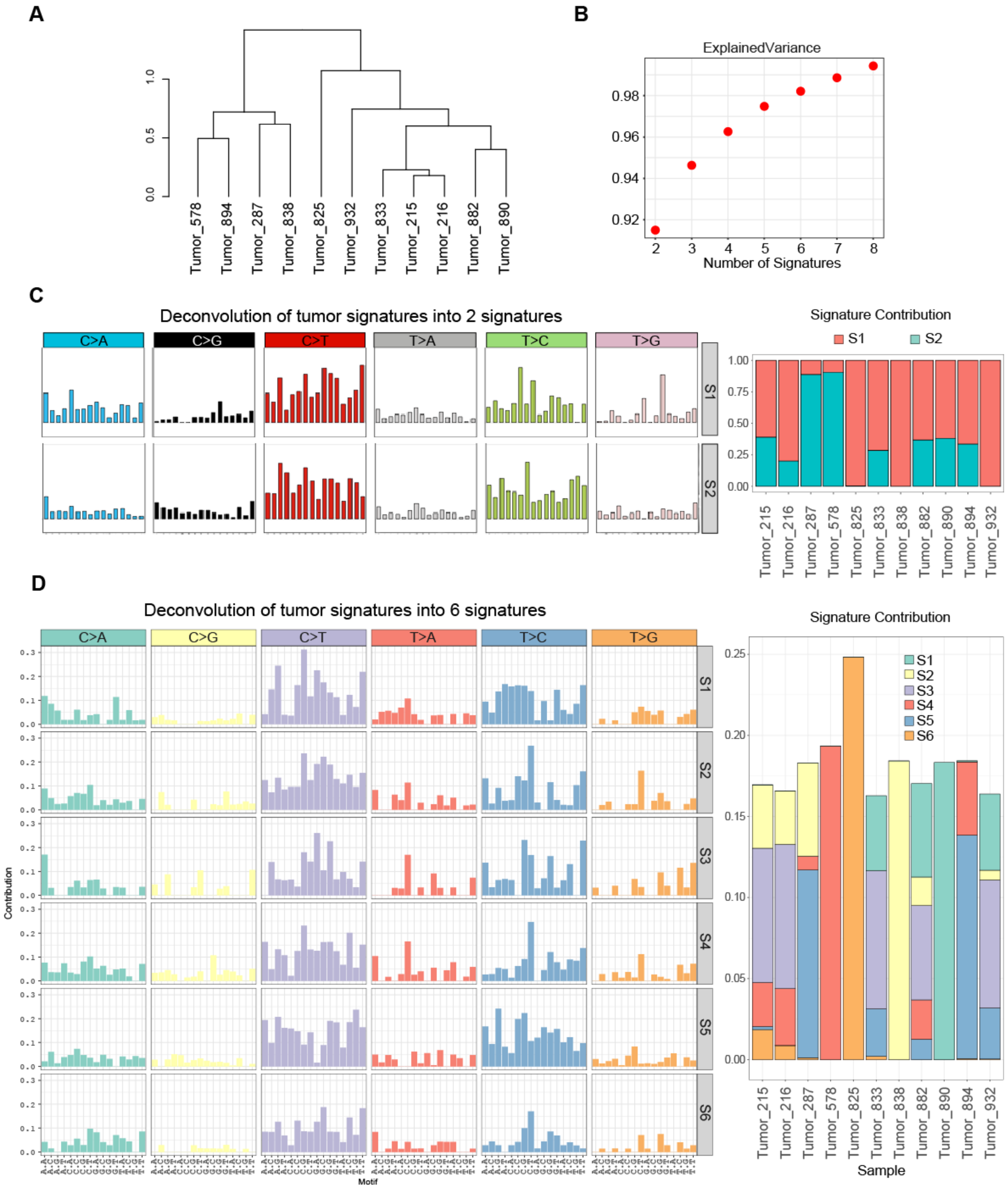

Figure S7: Mutational signatures in tumors from SKO mice. A: Clustering of 11 tumors by mutational burden. **B:** Graph showing explained variance with increasing numbers of *de novo* generated signatures. **C:** Deconvolution of data into two mutational signatures and contribution to the individual tumors. **D:** Deconvolution into six mutational signatures and contribution to the individual tumors.

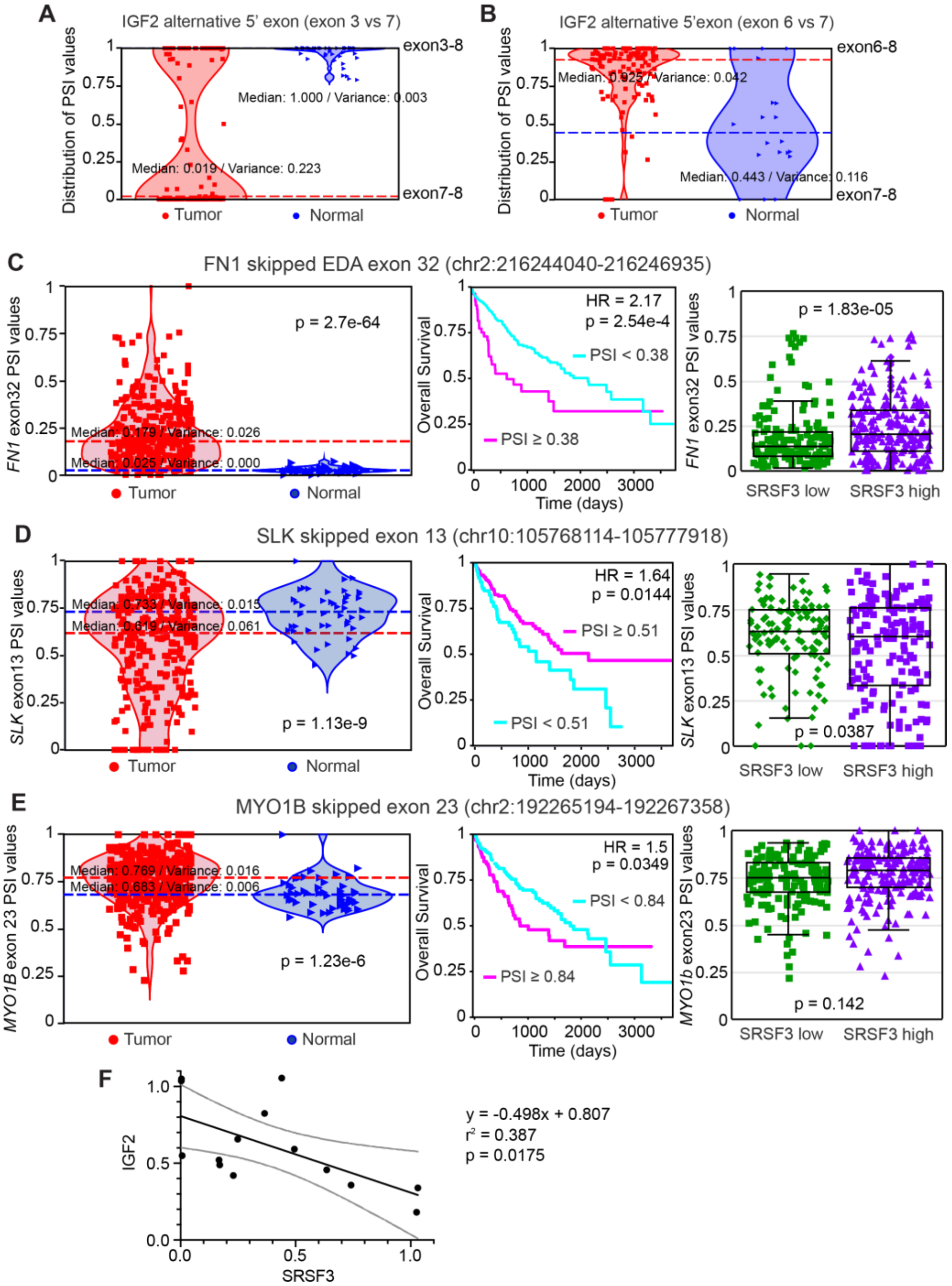

Figure S8: IGF2 alternative upstream exon usage in HCC and correlation of SRSF3-dependent splicing events with survival. A and B: Violin plots showing alternative splice site usage as percent spliced in (PSI) between exons 3-8 versus 7-8, and exons 6-8 and 7-8. Normal liver samples are shown in blue triangles, HCC in red squares. Median splice site usage and variance is indicated. **C:** Violin plot showing skipping or inclusion of the EDA exon 32 in the *FN1* gene on left. Statistical significance for difference in PSI values (p = 2.7e-64). Survival plot for patients with *FN1* exon 32 PSI < 0.38 (cyan) vs >= 0.38 (magenta) in middle. The hazard ratio (HR) is 2.17, p = 2.54e-4. Boxplot showing exon 32 inclusion in SRSF3 low (green) and high (purple) groups on right (p=1.83e-05). The boxplot shows median value, interquartile range and 95% confidence interval. **D:** Violin plot showing skipping or inclusion of exon 13 in the *SLK* gene on left. Statistical significance for difference in PSI values (p = 1.13e-9). Survival plot for patients with *SLK* exon 13 PSI < 0.51 (cyan) vs >= 0.51 (magenta) in middle. The hazard ratio (HR) is 1.64, p = 0.0144. Boxplot showing exon 13 inclusion in SRSF3 low (green) and high (purple) groups or right (p=0.0387). **E:** Violin plot showing skipping or inclusion of exon 23 in the *MYO1B* gene on left. Statistical significance for difference in PSI values (p = 1.23e-6). Survival plot for patients with *MYO1B* exon 23 PSI < 0.84 (cyan) vs >= 0.84 (magenta) in middle. The hazard ratio (HR) is 1.5, p = 0.0349. Boxplot showing exon 23 inclusion in SRSF3 low (green) and high (purple) groups on right (p=0.142). **F:** Correlation of IGF2 and SRSF3 protein levels in frozen HCC samples. Gray curves show 95% confidence interval.

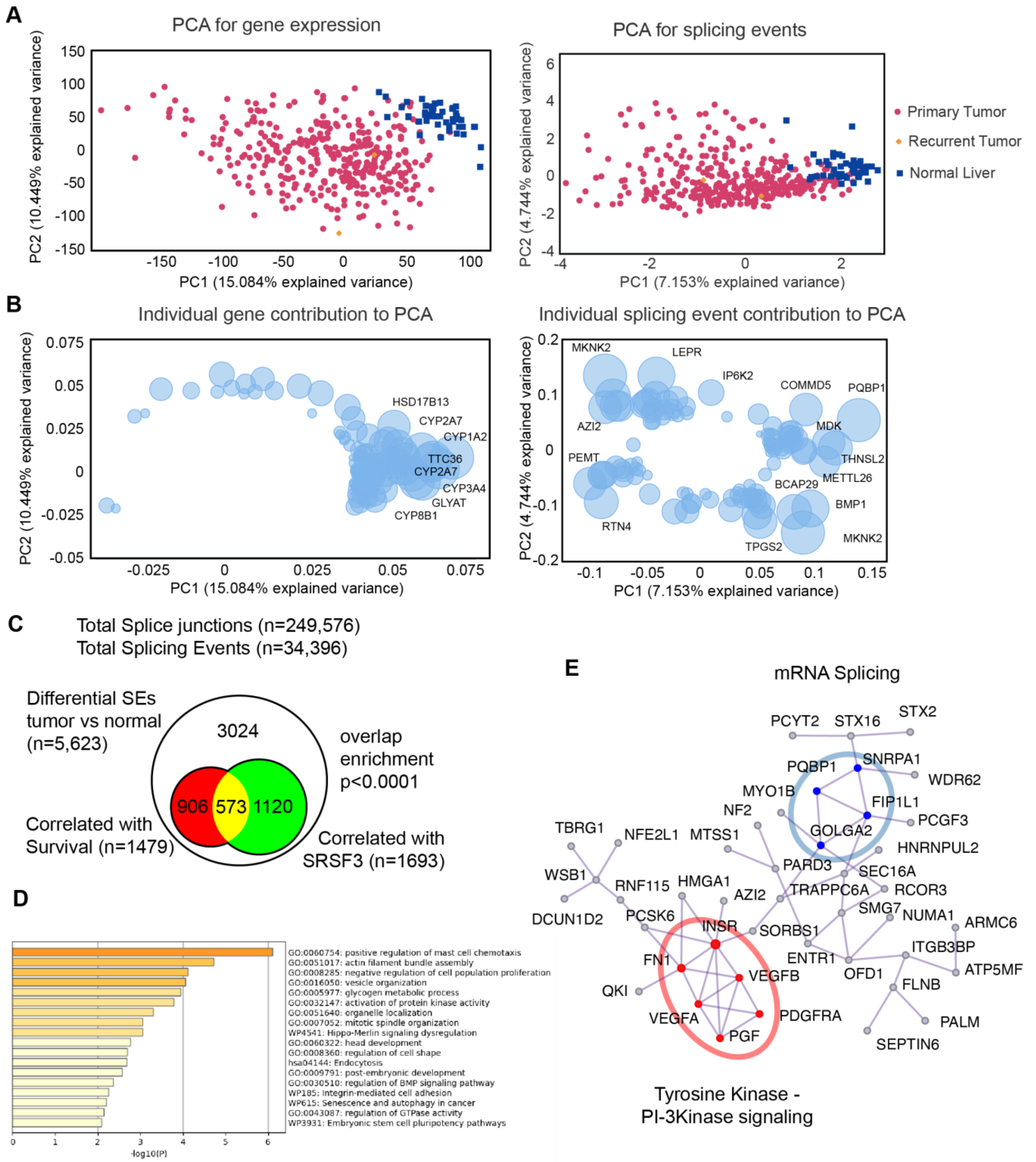

Figure S9: Changes in alternative splicing in the LIHC dataset. A: Principal component analysis of gene expression and splicing events in the LIHC dataset. Primary tumor samples are shown in red circles, recurrent tumors in yellow diamonds, and normal tissue in blue squares. The two major principal components are shown on the x and y-axes. **B:** PCA loading plots showing the contribution of individual genes or splicing events to the principal components. Size of the bubble indicates the size of the contribution. The genes corresponding to the largest bubbles are indicated. **C:** Venn diagram showing overlap of all differential splicing events associated with overall survival versus those correlated with *SRSF3* expression in the LIHC dataset. **D:** Pathway enrichment analysis for top 103 genes with ΔPSI > 0.1 that correlated with both survival and *SRSF3* expression in the LIHC dataset. Colors indicate relative significance. p-values are indicated on the x-axis. **H:** Protein-protein interaction network for the top 103 genes. Significant MCODE subnets for tyrosine kinase/PI-3Kinase signaling (red) and mRNA splicing (blue) are indicated.

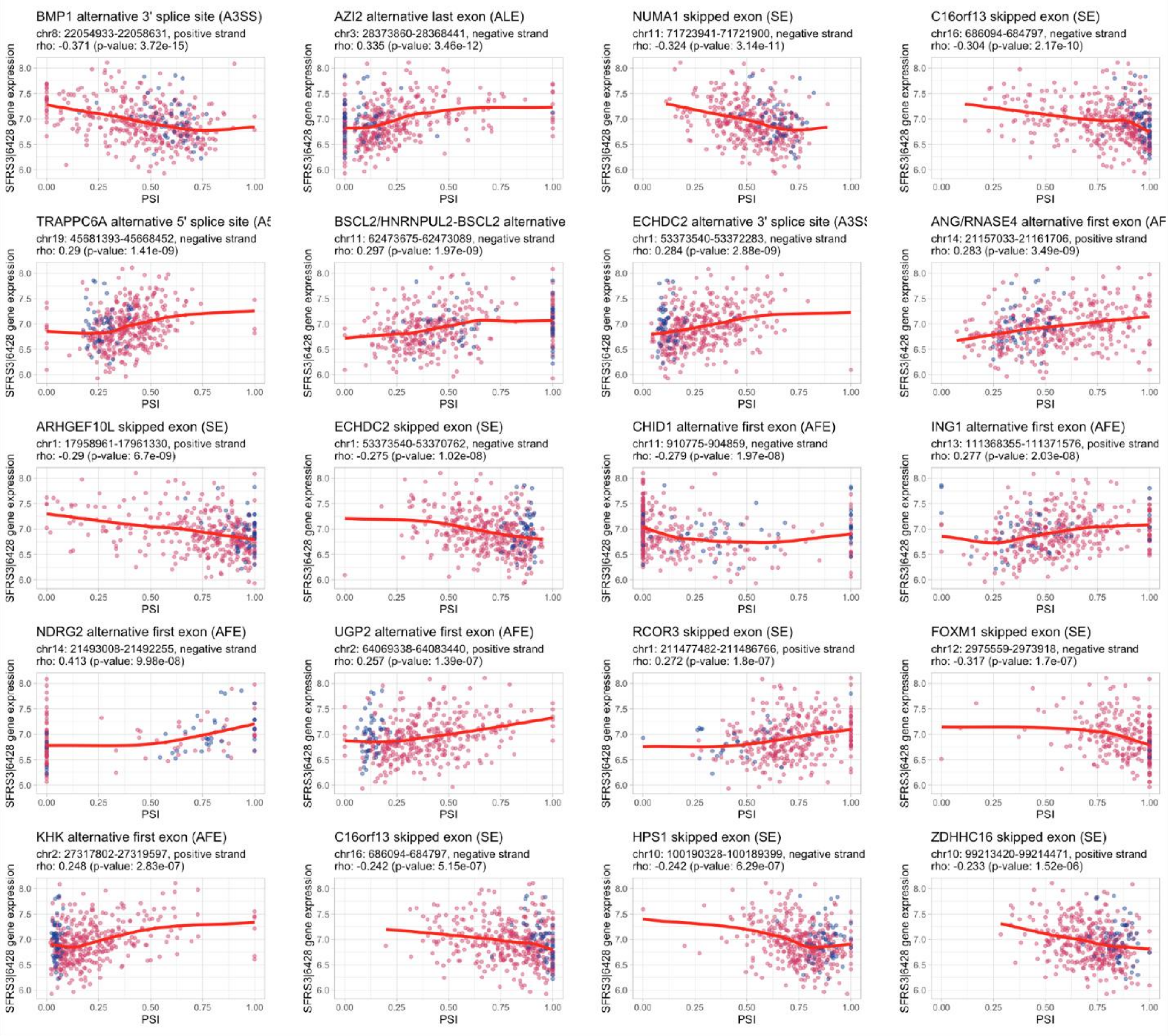

Figure S10. Correlation of top survival associated splicing events with *SRSF3* expression. Correlation between PSI of the individual splicing event and *SRSF3* mRNA expression. Chromosomal location of the splicing event is shown. Individual HCC samples are shown in red, normal samples in blue. Red lines are the loess curve fits for the correlations. Spearman rho correlation coefficient and p-value are shown.
